# Biofilms: Formation, Research Models, Potential Targets, and Methods for Prevention and Treatment

**DOI:** 10.1002/advs.202203291

**Published:** 2022-08-28

**Authors:** Yajuan Su, Jaime T. Yrastorza, Mitchell Matis, Jenna Cusick, Siwei Zhao, Guangshun Wang, Jingwei Xie

**Affiliations:** ^1^ Department of Surgery‐Transplant and Mary & Dick Holland Regenerative Medicine Program College of Medicine University of Nebraska Medical Center Omaha NE 68198 USA; ^2^ Department of Pathology and Microbiology College of Medicine University of Nebraska Medical Center Omaha NE 68198 USA; ^3^ Department of Mechanical and Materials Engineering College of Engineering University of Nebraska‐Lincoln Lincoln NE 68588 USA

**Keywords:** biofilms, formation, management, models, targets

## Abstract

Due to the continuous rise in biofilm‐related infections, biofilms seriously threaten human health. The formation of biofilms makes conventional antibiotics ineffective and dampens immune clearance. Therefore, it is important to understand the mechanisms of biofilm formation and develop novel strategies to treat biofilms more effectively. This review article begins with an introduction to biofilm formation in various clinical scenarios and their corresponding therapy. Established biofilm models used in research are then summarized. The potential targets which may assist in the development of new strategies for combating biofilms are further discussed. The novel technologies developed recently for the prevention and treatment of biofilms including antimicrobial surface coatings, physical removal of biofilms, development of new antimicrobial molecules, and delivery of antimicrobial agents are subsequently presented. Finally, directions for future studies are pointed out.

## Graphical abstract

This review first describes the formation of biofilms in various clinical scenarios and their clinical management. Then, the established biofilms models used in research and the potential targets for biofilms are discussed. Finally, this review highlights the emerging novel technologies for the prevention and treatment of biofilms.

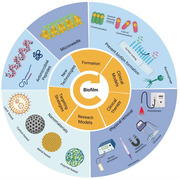

## 1 Introduction

### 1.1 Biofilm Formation

In the past few decades, the paradigm of microbiology has undergone a revolutionary shift. It was initially thought that microorganisms existed only as planktonic cells or floating cells.^[^ ^1^ ^]^ J. William Costerton, a Canadian microbiologist, changed that view in the late 1970s when he observed microbial aggregates which were known as biofilms.^[^ ^2^ ^]^ Biofilms are often described as microbial communities attached to material surfaces, formed by pathogens embedded in their own extracellular matrix (ECM) composed of several types of biopolymers, including extracellular polysaccharides, extracellular DNA, proteins, and lipids.^[^ ^3^ ^]^ Microorganisms in the biofilm account for <10% of dry mass, while ECM can account for >90%. The ECM forms the scaffold and typical 3D structure of the biofilm.^[^ ^4^ ^]^ In addition, the multiple functions of extracellular polymer matrices, including adhesion, intercellular aggregation, biofilm cohesion, water retention, barrier protection, and nutritional support, provide a wide range of advantages for biofilm formation.^[^ ^5^ ^]^

Biofilm formation goes through five steps.^[^ ^6^ ^]^ i) Individual plankton bacterial migrate and adhere to the surface. Under appropriate conditions, the attached bacteria start to form biofilms with a coating of a small amount of exopolymeric material. ii) Attached bacteria secrete extracellular polymeric substance (EPS) and stick to the surface, resulting in a conglomeration of bacteria and matrix production. iii) Biofilms fully develop by forming microcolonies and water channel structures, and become more layered. iv) Fully mature biofilms reach their peak cell density and function as 3D communities. v) Mature biofilms release bacterial microcolonies from the primary community, seeding new sites and spreading the infection. Such biofilms make it difficult for antibiotics to penetrate the matrix and kill the hidden bacteria.

### 1.2 Biofilms in Chronic Wounds

Chronic wounds often refer to wounds that fail to heal within a normal timeframe (usually within 1.5 months). Chronic wounds include a diverse array of different clinical scenarios such as surgical wounds, venous leg ulcers, diabetic foot ulcers (DFUs), and pressure ulcers.^[^ ^7^ ^]^ Due to the inherent pathophysiology of these wounds and the polymicrobial nature of the wound environment, chronic wounds often do not heal.^[^ ^8^ ^]^ Chronic wounds are an important and increasingly serious problem in today's medical care.^[^ ^9^ ^]^ In the U.S. 2% of the population potentially develop chronic wounds.^[^ ^10^ ^]^ The estimated cost for management of chronic wounds surpasses $50 billion annually.^[^ ^11^ ^]^

The unique micronutrients, wound surface, and exudate produced by the wound provides an ideal environment that supports three different phenotypic states for the growth of microorganisms: free‐floating (planktonic), attachment (sessile), and quasi‐sessile (first from the biological membrane separation of microbial aggregates or flocculation body).^[^ ^12^ ^]^ Sessile bacteria on the surface dynamically divide multiple times and form aggregate, forming microcolonies, which then merge to create dynamic entities called biofilms.^[^ ^13^ ^]^ Interestingly, the growth of microorganisms in biofilms follows the principle of “group selection” rather than “individual selection”.^[^ ^14^ ^]^ This seems critical for the managing infections associated with biofilms.^[^ ^15^ ^]^

In 2004, biofilms were conceptually reported to be the root cause of nonunion and long‐term infections seen in the majority of chronic wounds.^[^ ^16^ ^]^ In 2008, James and his colleagues strengthened this hypothesis by showing that 60% of chronic wounds contained biofilms.^[^ ^7^ ^]^ Lately, the role of biofilms in delaying chronic wound healing and increasing risk of infection has been further demonstrated by many studies.^[^ ^17^ ^]^ In 2012, Römling and Balsalobre showed that more than 80% of surgical site infections (SSIs) develop biofilms.^[^ ^18^ ^]^ However, the guidelines provided by the Centers for Disease Control and Prevention (CDC)’s SSIs prevention advisory committee do not mention biofilms.^[^ ^19^ ^]^ Biofilms are difficult for the host's immune system to defeat. The immune response to biofilms includes the stimulation and recruitment of polymorphonucleocytes and white blood cells, resulting in chronic inflammation that delays wound healing.^[^ ^20^ ^]^ Studies have shown the importance of biofilms in the persistence of wound infections, and the polymicrobial properties of biofilms are believed to be one of the chief factors that cause the recurrence of wound infections.^[^ ^21^ ^]^

**Figure 1:**
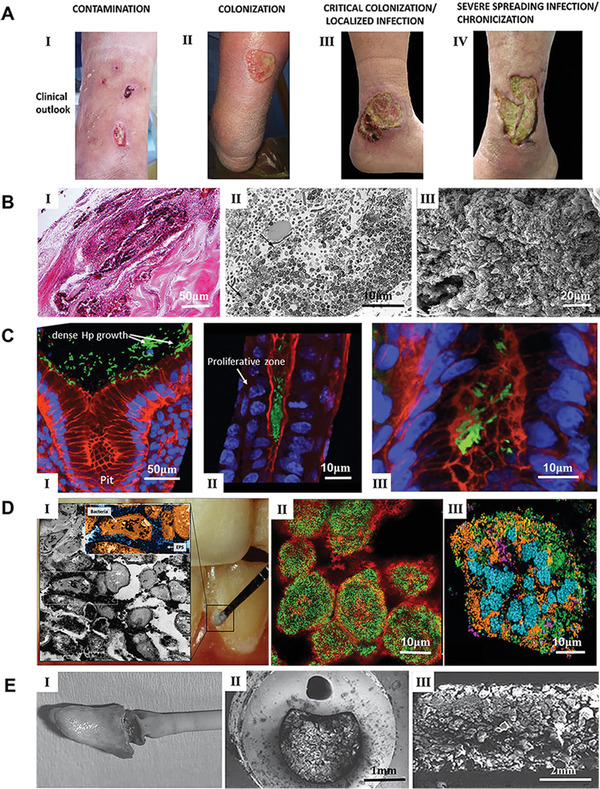
Representative bacterial biofilms within the clinical setting. A) The process of DFU wound infection, chronicization, and biofilm colonization. Reproduced with permission.^[^ ^28^ ^]^ Copyright 2019, MDPI. B) Representative images of biofilms on the full‐thickness burn wounds. I): Large collections of gram‐positive cocci form a biofilm on the surface of an ulcerated burn wound. Wound dressing remnants are present on the top left. II): Low power transmission electron micrograph of a mixed bacterial biofilm consisting of rods and cocci, some of which are degenerated (arrows). III): Scanning electron micrograph of the edge of an escharotomy site. The burn surface can be observed on the top right. A large collection of mixed bacteria with the typical appearance of a biofilm can be seen below the surface within dermal collagen. Reproduced with permission.^[^ ^45^ ^]^ Copyright 2010, Elsevier. C) In vivo evidence suggesting *H. pylori* biofilm formation in the gastric glands of humans. I): Large aggregates of *H. pylori* colonizing the surface of gastric glands; II): *H. pylori* aggregates colonizing the neck of gastric glands, with proliferative cells; III) colonies of *H. pylori* deep in the gland, in the vicinity of stem cells. *H. pylori* stained in green, actin stained in red and DNA nucleus stained in blue. Reproduced with permission.^[^ ^58^ ^]^ Copyright 2019, Frontiers Media S.A. D) Dental plaque architecture: The EPS matrix, spatial organization, and polymicrobial composition. I): Plaque biofilm from a caries‐active subject: microscopic image (inset) of plaque‐biofilm showing a selected area containing bacterial cells (highlighted in orange) enmeshed in EPS (in dark blue); the image was pseudo‐colored using Adobe Photoshop software for visualization purposes. II): Bacterial clusters (green) surrounded by EPS matrix (red) detected in mature mixed‐species oral biofilms formed in sucrose. III): Spatial organization of human dental plaque showing multiple clusters of varying sizes containing different microbial species. Reproduced with permission.^[^ ^71^ ^]^ Copyright 2018, Elsevier. E) I): A catheter was removed surgically that had been indwelling suprapubically for 6 months. Crystalline material completely covered the eyehole and balloon of the hydrogel‐coated latex catheter. II): A cross‐section of a silicone catheter that had been indwelling for 8 weeks. The image shows that the central lumen was occluded by crystalline biofilm. III): A longitudinal section of a silver‐hydrogel‐coated latex catheter that became blocked after 11 days in situ. Reproduced with permission.^[^ ^86^ ^]^ Copyright 2008, Springer Nature.

Around 15–25% of diabetic patients have DFUs in their lifetime.^[^ ^22^ ^]^ Infected DFUs are one of the most serious complications and potentially lead to lower limb amputations.^[^ ^23^ ^]^ Infection, poor healing, and ischemia are characteristics of DFUs.^[^ ^24^ ^]^ In fact, 80% of patients with diabetes develop biofilm‐infected foot ulcers before lower limb amputation.^[^ ^25^ ^]^ Infected DFUs are also associated with a higher mortality rate within 18 months. The interface between the host and microbes is critical in the development of DFUs.^[^ ^26^ ^]^ In DFUs, different bacteria are assembled into pathogroups with similar functions, which cause pathogenic and symbiotic bacteria to sustain chronic infections in biofilms.^[^ ^27^ ^]^ Such polymicrobial biofilms have been seen in both preclinical animal models and in patients with DFUs. They represent a major cause of delayed healing. The photographs shown in **Figure** **1**A illustrate the different clinical perspectives of DFU infection, including four stages: contamination, colonization, critical colonization of localized infection, and severe spreading infection/chronicization.^[^ ^28^ ^]^

In the 2010s, much clinical research demonstrated the presence of biofilms in chronic wounds. Neut et al. reported two case studies about nonhealing ulcers in people who have diabetes mellitus in 2011.^[^ ^29^ ^]^ Evidence of biofilms was shown in these patients’ DFUs using laser scanning confocal microscopy imaging. Malik et al. found biofilms in 67.9% of 162 patients with diabetic foot infection (DFI).^[^ ^30^ ^]^ In addition, Oates et al. visualized the biofilms in the tissues debrided from chronic wounds in diabetic patients using fluorescence in situ hybridization and scanning electron microscopy (SEM).^[^ ^31^ ^]^

It is believed that a single bacterial species do not cause biofilm formation during infection, especially in chronic wounds as microbes in biofilms are often polymicrobial.^[^ ^32^ ^]^ The interactions between microorganisms are complicated and significantly contribute to the pathogenesis of bacterial biofilm‐associated infections.^[^ ^33^ ^]^ These interactions can be antagonistic or cooperative. They often include rivalry for nutrients or collaborative mechanisms aiding their reciprocal growth in particular environments.^[^ ^34^ ^]^ The close‐contact between bacteria in biofilms enhances molecular communications between bacteria.^[^ ^34^, ^35^ ^]^ Bacteria communicate by diffusing molecules, such as the homoserine lactones or quinolones produced by Gram‐negative bacteria, or the short peptides produced by Gram‐positive cocci.^[^ ^36^ ^]^ In addition, this proximity allows horizontal gene transfer, facilitating resistance to antimicrobial agents and improving the survival of the biofilm. Mottola et al. examined 53 clinically derived *Staphylococcus* samples from DFU patients and found that biofilms are 10–1000 times more resistant to antibiotics than planktonic cells.^[^ ^37^ ^]^ In their studies, only two antibiotics including gentamicin and ceftaroline can destroy biofilms among the 10 antibiotics investigated. Bacterial biofilms are reported to offer high resistance to heavy metals and ultraviolet light as well. Other than bacteria, fungal species, in particular *candida*, were also found in biofilm‐containing DFU specimens.^[^ ^37^ ^]^

### 1.3 Biofilms in Burns

Burns extensively damage soft tissue, and, depending on the severity of the burn, may result in deep wounds and/or death.^[^ ^38^ ^]^ Medical treatment of burns has always been a difficult problem, which has given rise to many different methods to treat the damaged area.^[^ ^39^ ^]^ The presence of biofilms delays burn wound healing as it causes a continuous, low‐grade, inflammation, hindering the formation of granulation tissue and re‐epithelialization.^[^ ^40^ ^]^ One method for avoidance and management of burn infections is the administration of antimicrobial agents to kill bacteria.^[^ ^41^ ^]^

One of the biggest challenges facing burn clinics is the complication of bacterial infections within burn wounds, which can lead to more serious disease states, including sepsis.^[^ ^42^ ^]^ The lack of new antimicrobials, particularly those effective against biofilms, further exacerbates the challenge of treating drug‐resistant microorganisms in burn wound infections.^[^ ^43^ ^]^ In the immediate hours and days following a burn, Gram‐positive *Staphylococci* colonize the surface of the wound, as they are members of normal skin flora and are resistant to thermal damage.^[^ ^44^ ^]^ Other bacterial and fungal species (e.g., *Pseudomonas aeruginosa* (*P. aeruginosa*)) re‐colonize the burn wound surface usually within one week after the burn. Contamination of burn wounds by *P. aeruginosa* could cause invasion and sepsis, which may be fatal. Figure 1B shows representative images illustrating the characterization of biofilms in full‐thickness burn wounds.^[^ ^45^ ^]^ Figure 1B (I) shows a formed biofilm on an ulcerated burn wound. Wound dressing remains are noticed in the top left. Figure 1B (II) shows a transmission electron microscopy (TEM) image of the mixed bacterial biofilm made up of rods and cocci, part of which are degenerated indicated by arrows. Figure 1B (III) shows an SEM image of the edge of an escharotomy site. The burn surface is displayed in the top right. Massive accumulation of varied bacteria with the representative feature of a biofilm was observed beneath the surface within the dermis. The existence of biofilm in the burn wound poses challenges to managing burns. The best practice entails early excision and coverage of the burn wound to prevent the colonization of multiple bacteria.

### 1.4 Biofilms in Gastrointestinal (GI) Tract

The human GI tract extends from the esophagus to the rectum and includes the stomach, small intestine, and large intestine (colon). The GI tract contains a variety of microhabitats that are variously colonized by microorganisms based on the conditions of the microhabitat.^[^ ^46^ ^]^ Colonization gradients exist in the gastrointestinal tract, ranging from the scarcely populated esophagus and stomach to the densely colonized colon, where the luminal contents can accommodate as many as 10 ^12^ culturable bacteria/g.^[^ ^47^ ^]^

Many studies have shown the presence of both inflammation and microbial biofilms within the GI system.^[^ ^48^, ^49^ ^]^ The GI diseases associated with biofilms that satisfy these conditions include *Helicobacter pylori* (*H. pylori*) infections, Barrett's esophagus (BE), inflammatory bowel disease (IBD) (e.g., Crohn's disease and ulcerative colitis (UC)), and nasogastric (NG)/percutaneous endoscopic gastrostomy (PEG) tubes.^[^ ^50^ ^]^ The cause‐and‐effect relationship between local *H. pylori* biofilms and persistence in the host has been reported.^[^ ^51^ ^]^ *H. pylori* biofilms can be visualized directly in the gastric mucosa which are resistant to antimicrobial agents, making treatment difficult.^[^ ^52^ ^]^ Another GI disease, BE, is associated with localized nitrate reduction due to the biofilms of *Campylobacter* and *Veillonellas*, which could lead to metaplastic changes in esophageal squamous epithelial cells. Although important, studying a causal link between these bacteria and progression to BE has proved difficult.^[^ ^53^ ^]^ The microbiome related to IBD and the positive outcome of antibiotic therapy on these diseases have been described.^[^ ^54^ ^]^ However, as with diseases caused by other biofilms, patients may endure a “rebound effect” after the termination of antibiotic treatment because the bacteria not fully cleared by antimicrobial agents could regrow in the GI tract, likely resuming IBD symptoms.^[^ ^55^ ^]^ Indwelling medical devices associated with biofilms have been thoroughly described such as NG tubes and PEG tubes in neonatal and elderly patients. The microbial species associated with this phenomenon include *Enterobacteriaceae*, *S. aureus*, *lactobacilli*, and *Candida* spp., all of which have shown an increased resistance to elimination by antimicrobial agents in the form of biofilms as opposed to individual cells.^[^ ^56^, ^57^ ^]^ Therefore, due to the many different infections on these indwelling devices, their replacement becomes the last resort although undesired. Figure 1C shows the in vivo evidence of *H. pylori* biofilm formation in the gastric glands of humans.^[^ ^58^ ^]^ Figure 1C (I) shows colonized *H. pylori* on the surface of gastric glands. Figure 1C (II) shows colonized *H. pylori* aggregates on the neck of gastric glands, with proliferating cells, and Figure 1C (III) shows colonized *H. pylori* deep in the gland, close to stem cells. *H. pylori*, actin, and nuclear DNA were stained in green, red, and blue, respectively. In this study, *H. pylori* was stained with antibodies specific to the pathogen, confirming that the in vivo microbial aggregates were formed exclusively by *H. pylori*. The mechanism of the interactions between the biofilm community and the gastric mucosa in different niches of the gland could be useful for understanding the cause of serious diseases, such as gastric cancer and peptic ulcer. In particular, large aggregates of *H. pylori* growing in intimate contact with stem cells could generate potential damage to these cells due to the direct interaction with the bacteria.

### 1.5 Biofilms in Oral Cavity

Due to the warmth, high humidity, and rich nutrients, oral cavity offers a perfect environment for the growth of microorganisms.^[^ ^59^ ^]^ The complicated interplay between microorganisms, host, and diet can lead to the emergence of pathogenic oral biofilms.^[^ ^60^ ^]^ Oral biofilms can form on the surface of teeth or other dental surfaces and have shown to be an important virulence factor in a lot of oral infections.^[^ ^61^ ^]^

Bacteria can colonize on two types of surfaces within the oral cavity, including the hard surface of the teeth and the soft tissue of the oral mucosa. The teeth, tongue, gingival groove, hard and soft palates, cheeks, and tonsils all have favorable conditions for the growth of microbial colonies.^[^ ^62^ ^]^ These microbial aggregates are in the form of biofilms distinguished by their composition, coverage or matrix combination, and regulatory membranes covering the surfaces on which they are arranged.^[^ ^63^ ^]^ Due to changes in basic environmental conditions, the arrangement of resident oral microbiomes on diverse surfaces shows regional differences. Each niche provides a different optimal state and nutritional requirements for its hosted microbes. In this sense, the jaw, tongue, and hard and soft palates contain distinct bacterial components.^[^ ^64^ ^]^ In addition, the oral microbiome is highly dynamic due to the frequent contact between the oral cavity and the external environment.^[^ ^65^ ^]^ As a result, the oral microbiota has developed the ability to deal with difficulties that no other microbiome has encountered. The growth and activity of bacteria in the mouth are affected by feeding and preventing disease.^[^ ^66^ ^]^ In addition, microbial ecosystems are influenced by hygiene practices. However, oral microbial colonies with less sensitivity to disturbances undergo alterations related to health, diet, and age, along with steady variations in pH, redox potential, salinity, climatic conditions, and salivary water activity.^[^ ^67^ ^]^ Oral cavity bacteria are divided into 13 independent phyla, including *Acidobacteria*, *Actinobacteria*, *Bacteroidetes*, *Chloroflexi*, *Cyanobacteria*, *Deinococcus*, *Firmicutes*, *Fusobacteria*, *Proteobacteria*, *Spirochaetes*, *Synergistes*, SR1, and TM7.^[^ ^68^ ^]^

Bacterial biofilms are the primary cause of dental diseases. Dental caries are typically caused by biofilms, resulting in mineralized tooth tissue loss.^[^ ^69^ ^]^ Microorganisms in the mouth are necessary to cause caries, but that is not enough, because the formation of caries biofilm depends on the diet of the host.^[^ ^70^ ^]^ A sugar‐rich diet boosts the aggregation of EPS and simulates the agglomeration of acid‐producing/resistant microbiota, which is evidenced by microscopic images of plaque biofilms collected from the active site of caries that show bacteria within a rich EPS. (Figure 1D).^[^ ^71^ ^]^ Figure 1D (I) shows the plaque biofilm from a caries‐active subject, and the plaque‐biofilm containing bacterial cells enmeshed in EPS. Figure 1D (II) shows the bacterial aggregates embedded in EPS identified in hybrid‐species oral biofilms established in sucrose. Figure 1D (III) shows a human dental plaque with many aggregates of various sizes consisting of diverse microbial species. *Streptococcus mutans* (*S. mutans*) is a major bacterial strain associated with dental caries. Much research, including clinical, epidemiological, and animal studies, has shown that *S. mutans* is closely related to the disease, particularly in early childhood caries.^[^ ^72^ ^]^ One of the major adaptations that makes *S. mutans* an effective opportunistic pathogen in the oral microbiome is its special ability to use many different carbohydrates to produce EPS and acids, which allows it to easily assemble into biofilms. This ability includes mechanisms of stress resistance and bacteria ability. *S. mutans* obviously does not cause caries alone, its dynamic and collaborative interactions with multiple other organisms allow for the assembly of caries‐producing biofilms.^[^ ^73^ ^]^

### 1.6 Biofilms On/Within Medical Implants

Biofilms are commonly found on indwelling medical devices including catheters, heart valves, pacemakers, artificial joints, voice prostheses, and contact lenses.^[^ ^74^ ^]^ Biofilms may be formed out of a single or multiple microbial species, relying on the device and the duration it has been implanted for.^[^ ^75^ ^]^

There are two kinds of contact lenses, soft and hard which are classified based on building materials, disposal frequency, wear schedule, and design. Microbes can attach themselves to both types of lenses.^[^ ^76^ ^]^ The main types of microbes found on contact lenses include *E. coli*, *P. aeruginosa*, *S. aureus*, *S. epidermidis*, and species of *Candida*, *Serratia*, and *Proteus*.^[^ ^77^ ^]^ The extent of attachment to the lens is subject to the hydration status, substrate characteristics, electrolyte contents, bacterial species, and polymer types.^[^ ^78^ ^]^ Biofilms have been found by SEM on contact lenses of patients with diagnosis of keratitis due to the *P. aeruginosa* contamination. Contact lenses stored in cases may have more frequent biofilm growth. Lens storage cases have thus been believed to be a common source of contamination.^[^ ^79^ ^]^

Biofilms are commonly found on central venous catheters. The kind of biofilm, the location of the growth, and how pervasive the biofilm are all depend on the duration of the catheter insertion. For example, catheters that have been indwelling for fewer than ten days tend to form biofilms on the outer surface of the catheter, meanwhile long‐term (30 days) catheters form more biofilms in the lumen.^[^ ^80^ ^]^ Additionally, microbial growth could be influenced by the kind of fluid introduced through the central venous catheter. For instance, Gram‐positive bacteria (e.g., *S. epidermidis* and *S. aureus*) grow poorly in intravenous fluids, but gram‐negative aquatic bacteria (e.g., *P. aeruginosa*, *Enterobacter*, and *Klebsiella*) thrive in such fluids.^[^ ^81^ ^]^

Microbes that have adhered to heart valves and their surrounding tissues often generate biofilms, which produces a condition called prosthetic valve endocarditis. This kind of infection is most often caused by *S. aureus*, *S. epidermidis*, *Streptococcus* species, *Enterococcus*, gram‐negative *Bacillus*, and *Candida* spp.^[^ ^82^ ^]^ These microorganisms may derive from the endogenous flora on the skin or from other indwelling devices like central venous catheters or dental implants. Surgical damage during prosthetic valve implantation may also cause an accumulation of platelets and fibrin at suture sites, which provides an ideal milieu for bacterial colonization and subsequent biofilm formation.^[^ ^83^ ^]^

Most commonly, urinary catheters are constructed of silicon or latex, and are often employed during surgery to evaluate urine production.^[^ ^84^ ^]^ The catheter is inserted through the urethra and into the bladder. This kind of catheterization may be open or closed to the outside environment. In a catheter system open to the outside, urine is discharged at an open collection center, likely leading to higher chances of contamination and urinary tract infections (UTIs) in just a few days. In closed ductal systems, urine accumulates in plastic bags, thus minimizing the opportunities for contamination and resulting in lower rates of UTIs. Microbial contamination and biofilm formation on urinary catheters are most commonly from *E. coli*, *E. faecalis*, *S. epidermidis*, *P. aeruginosa*, and other bacteria.^[^ ^85^ ^]^ Figure 1E (I) shows an indwelling catheter after use for 6 months. After surgical removal, crystalline materials fully covered the eyehole and balloon of the latex catheter coated with hydrogel. Figure 1E (II) shows a cross‐section of an indwelling silicone catheter after application for 8 weeks, indicating the central lumen was blocked by crystalline biofilms. Figure 1E (III) shows a longitudinal section of a silver‐hydrogel‐coated latex catheter that was clogged after application for 11 days.^[^ ^86^ ^]^ These biofilms formed on the outer surface of the catheter around the balloon and catheter tip could cause trauma to the bladder and urethral epithelia. When the retained balloon is deflated, crystalline debris from the biofilms may fall off into the bladder and trigger stone formation.^[^ ^86^ ^]^ However, the major complication is obstruction of urine flow through the catheter likely due to the accumulation of crystalline material on the luminal surface. As a result, urine leakage often occurs along the outside of the catheter and patients would have urinary incontinence, leading to an increased need for care. In addition, the blockage of the catheter could cause retention of urine in the bladder and vesicoureteral reflux of infected urine. If the blockage is not detected and the catheter is not replaced, patients would suffer episodes of pyelonephritis and septicemia.

### 1.7 Clinical Detection of Biofilms

There are two main types of biofilm infections. One is related to biofilms in tissues (e.g., chronic wound infections, lung infection, and gastric *H. pylori* infection), and the other is medical devices associated biofilm infections (e.g., intravenous catheters, orthopedic alloplastic devices, endotracheal tubes, indwelling urinary catheters, and tissue fillers).^[^ ^87^ ^]^ Accurate detection or diagnosis of these biofilm infections is critical for their successful treatment (e.g., selection of appropriate antibiotic therapy). Here, we highlight the detection of biofilms in several important clinical scenarios.

For patients with chronic wound infections, it is important to detect the location of biofilms and the pathogen types. It is recommended to collect the biopsy tissue from the postdebridement wound bed for chronic wounds with suspected biofilm infections. Three major assays have been used for the biofilm diagnosis including morphology assay (e.g., tissue biopsy for histology and SEM and confocal laser scanning microscopy (CLSM) for detecting the location of biofilms), microbiology assay (e.g., bacterial culture for detecting the bacterial type) and molecular assay (e.g., 16S rRNA PCR for detecting the pathogen type). The advantages and drawbacks of each assay were listed in a recent excellent review article.^[^ ^87^ ^]^ For the gastric *H. pylori* infection, two major methods are currently used for its detection. One is an invasive test, which involves the use of endoscopy to observe mucosa and sample biopsies in multiple locations followed by histopathological analysis (a gold standard with 95% of sensitivity and 98% of specificity). Rapid urease test and culture and organism genotyping could be used to assist the detection as well. The other one involves noninvasive tests such as urea breath testing, stool antigen assay, and tests on plasma, blood, saliva, and urine. It is worth mentioning that GastroPanel representing a new‐generation test evaluates antibodies and pepsinogen I plus and pepsinogen II and gastrin‐17 in the plasma simultaneously with 94–95% of accuracy.^[^ ^88^ ^]^ For lung infection, examining sputum sample microscopically and culturing expectorants remain the major method for diagnosis because of its simplicity, quickness, and low cost.^[^ ^89^ ^]^ However, sputum examination may not be able to detect infections due to false negative results and contamination issues, more invasive methods can be used for sampling such as pulmonary endoscopy (e.g., bronchoscopy), transthoracic needle aspiration, and surgical biopsy of lung parenchyma. Similarly, culture, histology, nucleic acid test, and antigen testing allow the detection of suspected pathogens. In addition, many microbiologic assays (e.g., serum, nasopharyngeal swab, throat swab, urine, sputum, and body fluid) are available for diagnosis of lower respiratory tract infection.^[^ ^90^ ^]^

Patients with suspected infections associated with orthopedic implants, synovial fluid is collected for pathogen detection initially.^[^ ^87^ ^]^ Then, debridement is suggested if the aspirate with white blood cell count is larger than 25 000 per mm^3^.^[^ ^91^ ^]^ Three to six biopsies (less than 1 cm^3^) from peri‐implant tissue should be acquired based on the clinical practice guidelines.^[^ ^92^ ^]^ In addition, sonication of the explanted orthopedic implant or parts can assist the release of biofilms. The same pathogen appears in more than two culture specimens, confirming periprosthetic joint infection.^[^ ^93^ ^]^ SEM could also be used to directly visualize the biofilms on the surface of implants. For the suspected catheter‐associated infection, qualitative and quantitative blood cultures from vascular catheter and peripheral blood should be performed for diagnosis if the catheter is still in place.^[^ ^94^, ^95^ ^]^ For the removed catheter, the tip should be tested for a quantitative culture (threshold larger than 10^3^ CFU/mL) after sonication or vortex.^[^ ^96^ ^]^ Alternatively, a semiquantitative culture (threshold larger than 15 CFU) should be performed by rolling the catheter tip on an agar plate.^[^ ^97^ ^]^ It seems that most of the current methods for clinical detection of biofilm infections necessitate acquiring biopsies in an invasive way. Further studies are required to improve the detection of biofilm infections in different clinical scenarios such as new non‐invasive approaches for detecting biofilms in situ.^[^ ^98^ ^]^

### 1.8 Clinical Therapy

#### 1.8.1 Debridement

Debridement is widely used in clinics to treat different biofilms, which is performed by making use of mechanical destruction and/or chemicals.^[^ ^99^ ^]^ Debridement methods vary from sharp, specialized surgical debridement, to gentle mechanical debridement using curettes, fabric pads, douches, or ultrasound, to autolysis debridement using moisturizing dressings. Physical debridement is obviously the simplest and most effective method to eliminate biofilms, nonviable tissue, and foreign debris. While physicians have long known that debridement of carrion promotes healing, debridement can also remove bacteria that have colonized necrotic wounds and those growing in wound biofilms.^[^ ^100^ ^]^ Water irrigation techniques have been developed and are used for the removal of pathogen biofilms (e.g., SSIs, dental biofilms).^[^ ^101^ ^]^ High‐speed imaging has provided important insights into the fluid‐biofilm surface interactions, revealing that biofilms liquidize and spread across the entire surface despite the removal of a significant amount of biofilm from the region.^[^ ^102^ ^]^ The fluidization of biofilms contributes to the persistence of bacteria on the surface after water‐based removal and may result in the poor efficacy of treatment by rinsing and debridement for periprosthetic infections. Water‐based jets remain useful because antimicrobial therapeutics can be incorporated, thus the fluid can double as both a delivery device and a debridement device.^[^ ^103^ ^]^ Solutions or gels having preservatives (e.g., sodium hypochlorite or hypochlorite) are normally used for enzymatic/chemical debridement.^[^ ^104^ ^]^ Regardless of the removal method, clinical experience has shown that biofilms reform quickly within a day. Therefore, regular debridement is one of the key methods to removing biofilms. In addition, while slough is adjacent to underlying healthy host tissues, biofilms are often located on the very surface and thus likely respond better to milder debridement approaches like fabric pads, curettage, or chemical rinses.^[^ ^105^ ^]^ While debridement is intended to get rid of deactivated tissue and “repel” the biofilm to avoid reforming, it is only effective with the subsequent application of appropriate antibiotics and wound management products.^[^ ^106^ ^]^

#### 1.8.2 Topical Antimicrobial Therapy

Although there are some advances in anti‐biofilm therapies, especially for indwelling devices, the most common strategies are topical antimicrobial therapy. However, antibiotic resistance is on the rise worldwide and new treatments are urgently needed to address this challenge in healthcare.^[^ ^107^ ^]^ A large number of antimicrobials currently available (e.g., antibiotics, silver‐based products, iodine) could be confusing to healthcare professionals. Antibiotics should be administered carefully and with discrimination, only when infection is suspected clinically or confirmed by testing.^[^ ^108^ ^]^ Debridement eliminates part of the protective bacteria in biofilms provided by EPS, making the rest bacteria to increase their metabolic activity to rebuild. In this scenario, antibiotics originally developed to kill bacteria in the planktonic state and topical antimicrobials including silver, iodine, and polyhexamethylene biguanide become highly efficacious.^[^ ^109^ ^]^

The delivery system of an antimicrobial agent is just as critical as the specifically selected agent. The delivery system must interact optimally with the wound microenvironment.^[^ ^110^ ^]^ For instance, for wounds with biofilm exudation, a highly absorbable antimicrobial dressing should be applied following debridement. The application of sterile gauze or mesh is not appropriate because of insufficient exudate absorption capabilities. However, most topical antimicrobial products currently available have limited efficacy against biofilms.^[^ ^111^ ^]^ A careful combination of debridement, antimicrobial agents, and wound care products is the most effective strategy for the treatment of biofilms‐containing wounds.

Silver sulfadiazine remains the most popular drug with an excellent activity profile, low toxicity, and ease of use with marginal pain. Silver sulfadiazine is believed to act by inhibiting DNA replication and modifying cell membranes and walls. It is effective in killing both gram‐positive and gram‐negative bacteria; however, resistance has been revealed sporadically.^[^ ^112^ ^]^ Silver sulfadiazine is one of the therapies used to prevent and treat infections in second‐ and third‐degree burns. However, continued use of this treatment in large burns (> 50qc, total surface area, TBSA) fails.^[^ ^113^ ^]^ If infection is present or suspected, appropriate systemic antibiotic agents may be required. Cerium nitrate may be useful alongside silver sulfadiazine. Cerium nitrate has shown in vitro antibacterial activity and changes cell‐mediated immunosuppression after burns.^[^ ^114^ ^]^ Adding cerium nitrate to silver sulfadiazine may improve antimicrobial activity against gram‐positive and gram‐negative organisms and fungi. However, the use of cerium nitrate with silver sulfadiazine in clinics shows the same efficacy as silver sulfadiazine itself. Such a combination of drugs results in adherent eschar with satisfactory wound coverage before performing tangential excision.^[^ ^115^ ^]^

Povidone iodine ointment offers an effective combinatorial therapy combining the antimicrobial property with the moist environment required for wound healing. Even with the wide‐spectrum antibacterial activity, the application of povidone‐iodine‐based products for burn treatment remains debatable due to their toxicity to cells and delayed wound epithelial regeneration.^[^ ^116^ ^]^ Furthermore, povidone iodine ointments need to be applied four times per day to exhibit the largest antibacterial efficacy, which is a major disadvantage of this therapy when compared with other local antimicrobial drugs.^[^ ^117^ ^]^

At the same time, many other antimicrobial agents are also applied for topical antimicrobial therapy. Dakin's solution (0.025% sodium hypochlorite) is widely used in a variety of refractory wound types and is advised for burn wound management. It has a wide spectrum of bacterial killing and is effective against the clinical setting of methicillin‐resistant *Staphylococcus aureus* (MRSA), vancomycin‐resistant *Enterococcus* (VRE), and other antibiotic‐resistant bacteria.^[^ ^118^ ^]^ Although norfloxacin along with its silver salts has been formulated as topical creams due to its broad‐spectrum antimicrobial action, they require further investigations for the treatment of burns.^[^ ^119^ ^]^ MRSA strains are increasingly common as hospital pathogens, especially in burn wounds. Mupirocin has shown good efficacy in combating MRSA infections in vitro and in vivo. But there is a need to determine its safety and effectiveness in the management of burns greater than 20% TBSA. In addition, intranasal use of mupirocin ointment seems to minimize the chance of MSRA‐associated infections.^[^ ^120^ ^]^ Because of the emergence of gentamicin‐resistant bacteria, gentamicin cream should only be used for the management of gentamicin‐susceptible *P. aeruginosa* infected wounds and patients who show allergy to sulfonamides.^[^ ^121^ ^]^ Many clinical uses of bacitracin are for the prevention of gram‐positive bacterial infection in open spaces and the incorporation of neomycin and polymyxin B extends this antimicrobial effect to Gram‐negative bacteria.^[^ ^122^ ^]^

## 2 Biofilm Models for Research

### 2.1 In Vitro Biofilm Models

In vitro models are very important to the understanding of biofilms. These models can be classified as static or dynamic models according to the renewal of growth media and nutrients. The nutrient supply for static models is limited as the medium is not usually changed throughout the biofilm growth phase in a microplate. They are commonly used to assess biofilm formation and biomass accumulation. These models are cheap, simple, and repeatable. Therefore, they are widely used to evaluate biofilm growth dynamics and activity of anti‐biofilm compounds.^[^ ^123^ ^]^ Dynamic models, on the other hand, create an environment more similar to natural conditions for biofilm growth because there is a constant nutrient supply throughout the process. With the renewal of the culture medium and the removal of metabolites, it is possible to analyze the growth dynamics of biofilms over an extended period of time.^[^ ^124^ ^]^ In addition, more sophisticated systems facilitating flow displacement can be used to generate shear forces and mass transfer, thus creating environmental conditions similar to the in vivo environment.^[^ ^125^ ^]^ Currently, several commercially available static models are available to study in vitro biofilms. Microtitration‐based systems using 12, 24, or 96‐well plates are the most commonly used in vitro models. In these models, cultured biofilms are grown on the bottom and sides of the microtitration plate or placed on a given surface within the plate wells. These microplates provide a convenient and efficient method for comparing the biofilm‐forming ability of bacterial mutants^[^ ^126^ ^]^ or antibiofilm efficacy of multiple antimicrobial compounds or combined effects between them.^[^ ^127^, ^128^ ^]^ In addition, this method also enables multiple approaches to quantify biofilms by different staining methods (e.g., total biomass by using crystal violet and live cells by using 2,3‐Bis‐(2‐Methoxy‐4‐Nitro‐5‐Sulfophenyl)‐2H‐Tetrazolium‐5‐Carboxanilide (XTT) to get a more complete picture.^[^ ^128^ ^]^ It appears that biomasses are reduced more rapidly than live bacteria in the biofilm, depicting a picture that the biofilms matrix is first disrupted followed by the killing of bacteria hidden within biofilms.

**Figure 2:**
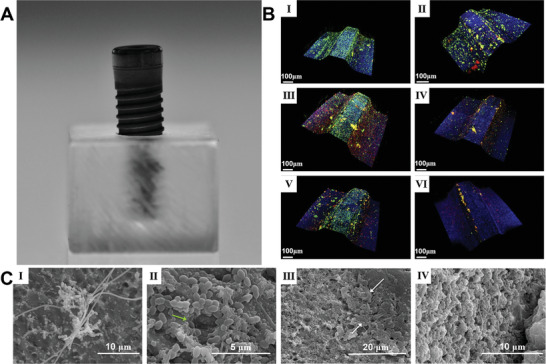
A representative in vitro biofilm model. A) Photograph showing a methacrylate stent with 10 mm wide and 7 mm high and an internal drilling with a diameter of 2.7 and 5 mm deep to support the implants in a fixed position allowing the exposure of the coronal third of the implant surface. B) CLSM Images obtained at 12 (I), 24 (II), 48 (III), 72 (IV), 96 (V), and 120 (VI) h of incubation of biofilms over whole dental implants which were stained using LIVE/DEAD BacLight Kit with live bacteria in green, dead bacteria in red, and implant surface in blue. C) SEM images showed biofilm growth from 48 to 120 h over whole dental implants. I): Biofilms after 48 h of incubation, with a complex morphology, in which *Fusobacterium nucleatum* formed networks with the adhered microcolonies of bacteria. II,III): Biofilms after 72 and 96 h of incubation, indicating the bacteria were in the expected larger stacks (growing masses of bacterial cells) and presence of broad channels (green arrow) and the cell mass and ECM surrounding bacteria in the biofilm (white arrows). IV): The biofilms after incubation from 72 to 120 h did not change in architecture. Reproduced with permission.^[^ ^129^, ^130^ ^]^ Copyright 2019, Wiley‐VCH.

To examine the biofilm formation on medical devices, Sanz et al. developed in vitro biofilm models by growing six bacterial strains on titanium implants for different durations (e.g., 12, 24, 48, 72, 96, and 120 h) (**Figure** **2**).^[^ ^129^ ^]^ Figure 2A shows a photograph of a methacrylate stent, 10 mm wide by 7 mm high, with internal boreholes 2.7 mm in diameter and 5 mm deep, used to fix and support the implant, displaying the coronal third of the implant. Figure 2B shows CLSM images of the entire dental implant obtained at 12 (I), 24 (II), 48 (III), 72 (IV), 96 (V), and 120 (VI) h in biofilm culture using LIVE/DEAD BacLight kit. Live bacteria, dead bacteria, and implant surfaces can be distinguished very clearly. Figure 2C shows SEM images of biofilm growth on the entire dental implant from 48 to 120 h. Image I shows a complex morphological biofilm after incubation for 48 h, in which *Fusobacterium nucleatum* forms a network with adherent bacterial microcolonies. In images II and III, bacteria and extensive channels were observed at 72 and 96 h in the anticipated large masses. The ECM (white arrow) surrounding bacteria in cell clusters and biofilms could be observed. Between72 and 120 h, the structure of biofilm remained the same, as shown in image IV. In general, the result showed the bacteria colonies were formed on implants rapidly, and biofilms matured by 96 h and displayed various ratios between live and dead cells determined by their location. Live bacteria were concentrated at the peaks of the threads. There is a fluctuation over time in terms of the densities of every colony with maximum values reached at 96 h.

### 2.2 Ex Vivo Biofilm Models

Several methods have been used to assess the infectious processes associated with biofilm formation. The development of ex vivo biofilm models has bridged in vitro models and animal models. In vitro models are not considered reliable due to the absence of significant host‐related biological factors. Animal models are often costly. Ex vivo models were proposed to bring experimental conditions closer to those observed in the host, once in vitro methods can be modified to simulate situations that happen in vivo.^[^ ^130^ ^]^ An important advantage of using ex vivo models is the possibility of reducing the number of animals used in research, which reduces the cost of maintaining experimental animals while respecting animal welfare. It is therefore important to stress that these models should prioritize the use of by‐products from abattoirs to ensure optimum use of animals for human consumption. However, ex vivo models aren't without their significant disadvantages. They lack the interaction of the host immune system with biofilms, and may lack competing microbiota present at different anatomical sites, and the insufficient fluid flow observed in in vivo studies.^[^ ^131^ ^]^

**Figure 3:**
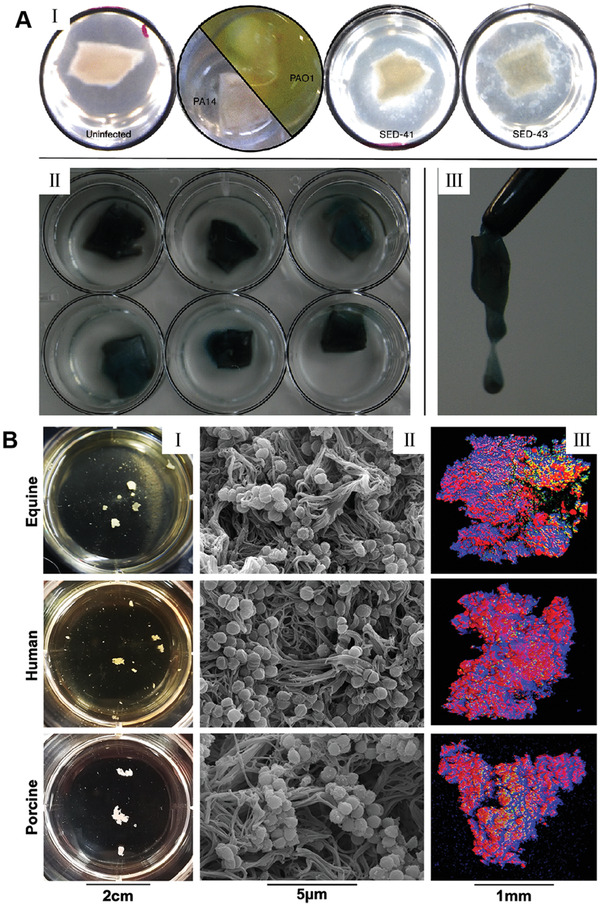
Two representative ex vivo biofilm models. A) An ex vivo lung model to study bronchioles infected with *P. aeruginosa* biofilms. I): EVPL in situ in ASM at 19 h post‐inoculation. Uninfected bronchiolar tissue retained its normal appearance: a pinkish‐white square with no noticeable degradation, surrounded by clear ASM. The laboratory strain PA14 did not show visible growth either on the tissue or in the surrounding ASM at this early stage; PAO1, in contrast, had grown extensively in the liquid ASM surrounding the tissue (green‐yellow pigmentation due to production of pyoverdine) but did not yet show any noticeable growth on the tissue itself‐note pinkish‐white square of tissue sitting in the liquid bacterial culture. In contrast, CF isolates of *P. aeruginosa* (e.g., SED‐41 and SED‐43) showed growth as frond‐like aggregates on and connected to the cubes of tissue, very different from the dense planktonic growth of PAO1. II) By 4 days post‐inoculation, CF isolates of *P. aeruginosa* had grown to a high density on EVPL. The image shows three replica infections of SED‐41 (top row) and SED‐43 (bottom row) after washing the tissue with phosphate‐buffered saline to remove non‐adhering cells: a coating of sticky *P. aeruginosa*, with blue‐green pigmentation (pyoverdine and pyocyanin), was left behind. III) These biofilms were noticeably mucoid (e.g., SED‐41). Reproduced with permission.^[^ ^135^ ^]^ Copyright 2016, Microbiology Society. B) *S. aureus* forms macroscopic biofilm aggregates in the synovial fluid of several different species. Equine, human or porcine synovial fluid was infected at 1×10^6^ CFU/mL with *S. aureus* (ATCC25923) and incubated overnight at 37 °C in a microaerophilic chamber on a shaker at 120 rpm to mimic the joint environment. I): Macroscopic biofilm aggregates were observed in synovial fluid in all three species and photographed. II): Aggregates were removed from the synovial fluid, fixed, dehydrated in ethanol, sputter coated, and imaged with an SEM, showing bacteria nested within a polymeric cord‐like ECM. III) Aggregates were stained with WGA in blue for carbohydrates, SYTO9 in green for nucleic acids, and SYPRO in red for proteinaceous content. 3D CLSM images were reconstructed by sequential Z‐stacking and tile scanning with Velocity software. Reproduced with permission.^[^ ^136^ ^]^ Copyright 2019, PLOS.

The characteristics of an ideal ex vivo biofilm model should include: 1) the use of by‐products from animal slaughter or surgery (human or animal), which reduces research costs and minimizes the use of healthy live animals; 2) easy control of microbial growth (disinfection or sterilization) to reduce microbial competition and allow microbial growth for research; 3) easy maintenance of tissue viability, which will reduce the cost of enrichment media or other reagents; and 4) the possibility of performing different analyses, including microbiological quantitative, microscopy and molecular analyses, to obtain more complex results.^[^ ^132^, ^133^, ^134^ ^]^ For a notable example, Harrison and Diggle demonstrated an optimized ex vivo model of cystic fibrosis (CF) lung infection by culturing pig bronchiolar tissue in artificial CF mucus.^[^ ^135^ ^]^ They focused on the formation of *P. aeruginosa* biofilms. As shown in **Figure** **3**A, the CF isolates established biofilms on bronchiolar tissue much faster and more specific than the laboratory isolates. Figure 3A (I) shows the ex vivo pig lung tissue localized in an artificial sputum medium 19 h after inoculation. The uninfected bronchioles retain their normal appearance as pink and white squares, without apparent degeneration, surrounded by clear ASM. At this early stage, the laboratory strain PA14 showed no visible growth in tissue or in surrounding ASM. In contrast, PAO1 grew dramatically in the fluid ASM surrounding the tissue but did not yet exhibit any significant growth on the tissue itself. But CF isolates of *P. aeruginosa* grew as leaf‐like aggregates linked to tissue cubes, showing a significant difference when compared with the dense planktic growth of PAO1. Figure 3A (II) shows CF isolates of *P. aeruginosa* (three replicates of SED‐41 and SED‐43 each) grew to high density on EVPL 4 days after inoculation. Figure 3A (III) shows these biofilms were distinctly mucilaginous. There was no visible *P. aeruginosa* PA14 growth either on the tissue or in the surrounding ASM for 19 h after inoculation. When culturing *P. aeruginosa* PAO1 and the CF isolates for the same period of time, there was visible bacterial growth. Even PAO1 grew avidly in the ASM surrounding the tissue, there was no noticeable growth on the tissue surface during the initial stage. On the contrary, after inoculation for 19 h, the CF isolates established frond‐like agglomerates attached to the tissue surface without detectable cloudiness of the adjacent liquid medium. These findings were in line with ex vivo pig lung tissue, and therefore provide a practical model environment for the lung‐adapted clones. In another study, Schaer et al. showed that *S. aureus* biofilm aggregates were established among all tested species after infection for 24 h (Figure 3B (I)).^[^ ^136^ ^]^ SEM images suggested that biofilm aggregates displayed almost identical morphological features in equine, porcine, and human synovial fluids. *S. aureus* encased in a polymeric, cord‐like ECM was noticed in every species (Figure 3B (II)). 3D reconstruction of confocal microscopy images indicated biofilm aggregates from synovial fluid showed a mixed protein stained with SYPRO in red, carbohydrate ECM stained with wheat germ agglutinin (WGA) in blue, and nucleic acid/bacterial stained with SYTO9 in green distributed through the aggregate in each species (Figure 3B (III)).

### 2.3 In Vivo Biofilm Models

Although this is a rapidly developing area of research, much about biofilm formation and behavior remains unknown, especially in the in vivo situations. Due to the pressing need for new chronic wound therapies, understanding the complexities of biofilm‐infected wounds is extremely important.^[^ ^137^ ^]^ Studies in understanding the interaction between biofilm properties and host inflammation are important to ameliorate this body‐of‐knowledge. Especially, the interplay between bacteria and host, namely the wound bed itself, accounts for part of the delineating characteristics of chronic wounds. This interaction cannot be assessed by in vitro biofilm models and analyses. Even if in vitro studies yield basic knowledge on biofilm resistance and survival mechanisms (e.g., biofilm inhibition of proliferation of keratinocytes), understanding the diverse and complex interactions between biofilms and wound‐healing pathways is difficult from in vitro studies alone.^[^ ^138^ ^]^

The absence of good in vivo models makes it hard to accurately simulate clinically wound biofilms. Human studies are logistically difficult and ethically impossible, which makes animal models the only viable substitute for systematically regulating clinically related biofilms. The use of animal models enables many experimental and analytical iterations that human research cannot afford, while enabling a closer approximation of biofilm‐host interactions which cannot be obtained in in vitro models.^[^ ^139^ ^]^ In addition, in vivo modeling allows for a direct understanding of the parallel pathways and mechanisms in human biofilm infections, and the translation of such information may guide more clinical studies.^[^ ^140^ ^]^ Thus, efficacious in vivo models are expected to inform more scientific understanding of biofilms, as well as offer a basis and method for systematic examination of biofilm‐infected wounds in an accurate and quantitative method.^[^ ^141^ ^]^

**Figure 4:**
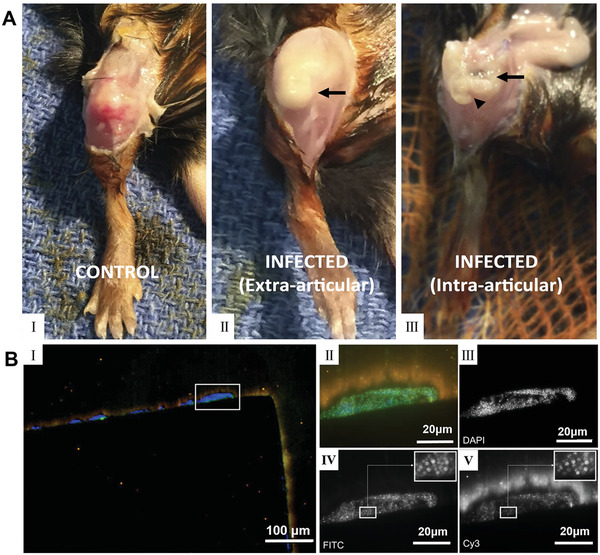
Two representative in vivo biofilm models. A) Photographs showing right knees of C57BL/6 mice after surgical implantation for 6 weeks. I): In the control animal, anatomical structures were preserved, with clear visualization of the patella and patellar tendon. II): In the animal infected with *S. aureus*, the patella was difficult to identify on superficial dissection. The underlying joint capsule (arrow) was distended because of being full of purulent, foul‐smelling, yellow material. III): Upon entering the joint, the proximal aspect of the tibia was fragmented and friable. The implant (arrowhead) was found within soft bone, was grossly loose, and was covered with yellow intra‐articular material (arrow). Reproduced with permission.^[^ ^143^ ^]^ Copyright 2017, The Journal of Bone and Joint Surgery, Inc. B) Fluorescence in situ hybridization (FISH) of in vitro grown *S. aureus* LS1 biofilms on a polytetrafluoroethylene catheter. The section was hybridized with the pan‐bacterial probe EUB338FITC (green), *S. aureus* specific probe SAUCy3 (yellow), nonsense probe NON338 (magenta), and nucleic acid was stained with DAPI (blue). I): Overview of the biofilm located on the outside of the catheter, showing multilayered cocci with strong fluorescence signals. II): High magnification image of boxed area in (I) showing merged image of all channels, indicating multilayered cocci with strong FISH signals. III–V): Black and white images of the single fluorescence channels showing nucleic acid staining with DAPI (III), pan‐bacterial probe EUB338FITC (IV), and *S. aureus* specific SAUCy3 signals (V). Note that all bacteria stained with EUB338 also show signals with probe SAUCy3. Reproduced with permission.^[^ ^144^ ^]^ Copyright 2017, Elsevier.

Another useful in vivo model is associated with biofilms formed around medical implants. The formation of biofilms renders conventional antibiotics and immune responses ineffective.^[^ ^142^ ^]^ Bostrom et al. used C57BL/6 mice with implantation of a unilateral proximal tibial graft and intra‐articular injection of *S. aureus* Xen 36 to establish an in vivo periprosthetic joint infection model.^[^ ^143^ ^]^ Autopsy of the infected knees in animals that underwent euthanasia at week 2 showed deformed soft‐tissue planes, pyogenic intra‐articular material, peri‐implant bone erosion, and a loose implant (**Figure** **4**A). At week 6, autopsy of the euthanized animals displayed extensive bone impairment and a loose implant. Contrarily, the controls showed an integration between bone and implant. In a different study, Van de Vyver et al. built a vascular graft infection model that closely mimicked the human environment by inserting a catheter into the right carotid artery of mice and inoculating 8 different *S. aureus* strains intravenously.^[^ ^144^ ^]^ The fluorescence in situ hybridization (FISH) analysis was applied to detect ribosomal RNA on the surface of vascular grafts in order to provide information regarding the differences in vivo and in vitro biofilm formation. Biofilms formed on a catheter in vitro were compared with those in the mouse infection model. FISH analysis of the in vitro‐grown biofilm on the catheters indicated the formation of multilayer biofilms on the external surface with a high number of bacteria dictated by bright FISH signals (Figure 4B). Moreover, FISH analysis of in vivo and in vitro catheters validated the formation of multilayered homogeneous biofilms in all studied samples. In the in vivo model, bacterial communities were mainly present at the blood‐graft contact site (e.g., the luminal surface of the catheter). Significant differences were observed in the structure of biofilms grown in vivo compared to in vitro biofilms, in which a large number of host cells fused with the matrix and bacteria. The number of FISH positive bacteria differed from FISH signal strength and was lower in catheters from in vivo models. Low FISH signals from bacteria in the body can be attributed to stationary or stationary patterns and may lead to increased resistance to antibiotic therapy in clinical settings. In addition, the static growth patterns in these biofilms led to decreased susceptibility to the immune system. These results highlight the relevance of the in vivo vascular graft model, because it more closely represents a true in vivo situation where bacterial metabolism is influenced by the immune system and blood flow.

## 3 Potential Targeting Strategies in Combating Biofilms

**Figure 5:**
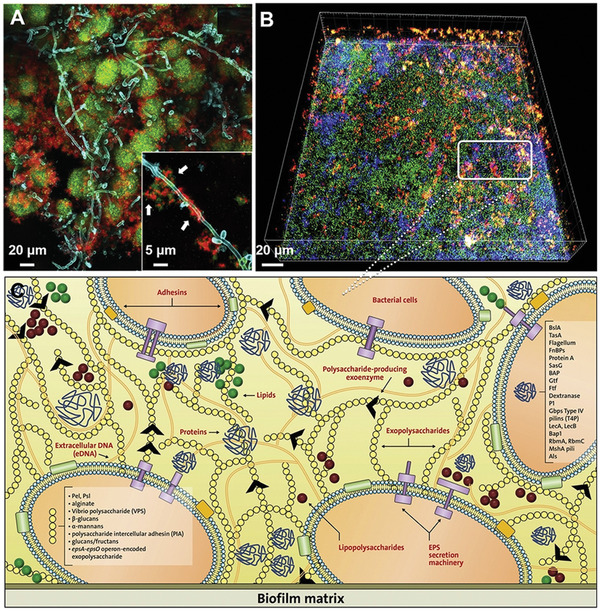
Compositions and functions of biofilm matrix in structured microbial communities. A) confocal fluorescence images of developed cross‐kingdom dental biofilms within ECM (red); inset shows *Streptococcus mutans* (green)‐*Candida albicans* (cyan) interactions mediated by ECM (white arrows). B) 3D reconstruction of CLSM images of in vitro oral biofilms after matrix staining. C) A schematic representation of the main components of the biofilm matrix and their functions. The biofilm matrix consisting of a wide array of functional biomolecules serves as a scaffold for structural support and a dynamic milieu that provides varying chemical and physical signals to microbial communities, promoting a biofilm lifestyle. Reproduced with permission.^[^ ^145^ ^]^ Copyright 2020, Elsevier.

A greater knowledge of the molecular compositions and functions of biofilms would be helpful in finding reasonable methods for prevention and treatment. **Figure** **5** shows the compositions and functions of biofilms in structured microbial communities.^[^ ^145^ ^]^ Figure 5A shows fluorescent images of formed cross‐kingdom dental biofilms within ECM, and the inset indicates ECM‐mediated interactions between *Streptococcus mutans* and *Candida albicans*. Figure 5B shows a 3D reconstruction CLSM image of an in vitro oral biofilm after matrix staining. Figure 5C shows a diagram of the major compositions and functions of biofilm matrix. It is made up of a diverse set of structures and molecules. It acts as a scaffold for structural support as well as a layout to facilitate various physical and chemical cues amongst the microbial community to promote the adoption of a biofilm lifestyle. Biofilms hold the ability to create distinct microenvironments with exclusive physical, chemical, phylogenetic, genotypic, and phenotypic heterogeneities. Earlier therapeutic approaches to treat bacterial infections by targeting individual cells had limited success. Currently, we have a better understanding of biofilm biology. Microbial biofilms represent a dynamic self‐constructed ecosystem within a matrix containing a highly heterogeneous and compartmentalized milieu, and more effective antibiofilm therapies probably need to target the complete microenvironment, as well as the individual cells within. Based on the compositions and functions, four potential targeting strategies could be used for the prevention and treatment of biofilms in theory.

### 3.1 EPS‐Targeting Strategies

The EPS composition in different biofilms shows temporospatial variations, which is subject to the strain of microorganism, regional mechanical shear force, substrate readiness, and host environment.^[^ ^146^ ^]^ EPS enhances microbial adhesion to surfaces, intercellular adhesion, and cell aggregation, and acts as a 3D scaffold to protect against host effectors and antibacterial therapy.^[^ ^147^ ^]^ In addition, EPS matrices can change chemical and nutritional gradations and map the pathogenic environment (e.g., pH and hypoxia), which contributes to important virulence properties.^[^ ^148^, ^149^ ^]^ Therefore, the EPS matrix is a favorable target for the destruction of biofilms, as doing so would decompose bacteria and degrade the pathogenic environment. Anti‐EPS strategies include inhibition of EPS production, prevention of microbial adhesion to the EPS matrix by inhibiting EPS adhesion, or degradation of EPS in established biofilms. For instance, DNase is effective in cleaving extracellular DNA (eDNA) of bacterial biofilms.^[^ ^150^ ^]^

### 3.2 Inducing Biofilm Dispersal

Biofilm dispersal is regulated and involves the degradation of EPS. Artificially triggering this reaction is the subject of many research strategies aimed at promoting the self‐decomposition of biofilms.^[^ ^151^ ^]^ In most cases, these methods postulate that dispersed bacteria have reverted to their more metabolically active planktonic state, which makes them more susceptible to conventional antibiotics.^[^ ^152^ ^]^ In addition, the released dormant cells would also lose protection due to their association with biofilm communities and structural tissues.^[^ ^153^ ^]^ Despite their dispersed state, it is still critical to dose dispersed or exogenous EPS bactericides with systemic antibiotics in a clinical setting to avoid recolonization, bacteremia, or sepsis.^[^ ^154^ ^]^

### 3.3 Metabolic Interference

There is considerable interest in the use of exogenous amino acids for biofilm therapy, and some amino acids have been demonstrated to influence biofilm metabolism and maturation.^[^ ^155^ ^]^ L‐arginine can be used as a substrate for arginine decomposition bacteria (such as *Streptococcus gordonii* (*S. gordonii*)) to produce alkali. It has been proved clinically that L‐Arg can neutralize acid and regulate the pH homeostasis in oral biofilm.^[^ ^156^ ^]^ Treatment with L‐Arg of polymicrobial biofilms containing *Streptococcus mutans (S. mutans)*, *S. gordonii*, and *Actinomycetes endrogeni* inhibited the growth of *S. mutans* and led to a significant decrease in insoluble EPS as well as changes in the structure of the biofilm. Other than pH regulation, L‐Arg can also inhibit genes associated with the generation of insolvable EPS and bacteriocins in *S. mutans*, meanwhile promoting the production of hydrogen peroxide (used against *S. mutans*) by *S. gordonii*. L‐Arg lowered the total biomass and rearranged EPS structure in *S. gordonii* biofilms. It also destabilized multiple species oral biofilms, thereby reducing the viability and increasing sensitivity to cetylpyridinium chloride.^[^ ^157^ ^]^ Another amino acid, L‐methionine (L‐Met), has also been recognized as an encouraging supplemental therapy for treating *P. aeruginosa* biofilms, as it triggered decomposition and increased sensitivity to ciprofloxacin in a mouse model of chronic pneumonia, and improved survival in infected mice.^[^ ^158^ ^]^ This activity is ascribed to the upregulation of four different DNase genes. Activation of these genes leads to the enzymatic destruction of eDNA in the EPS matrix, although the exact pathway that regulates this reaction has not been determined. Interestingly, L‐Met has been identified after screening for the activity of D‐amino acids and L‐amino acids against *P. aeruginosa* biofilms. Because of the versatility of amino acids used among bacterial species, a single amino acid is not likely to have the same function across bacterial species. Isoleucine has been shown to induce the expression of antimicrobial peptides (AMPs) that help inhibit bacteria and biofilms.^[^ ^159^ ^]^ Therefore, the essential role of amino acids in bacterial metabolism and host defense should not be discounted from further research for therapeutic strategies.^[^ ^160^, ^161^ ^]^

Another approach to metabolic interference stems from evidence that has shown iron metabolism to be critical for the biofilm formation of several pathogenic microbes.^[^ ^162^, ^163^, ^164^, ^165^, ^166^ ^]^ Iron access is critical for establishing infection with pathogens, and epithelial cells containing mutations of the CF transmembrane conductance regulator ΔF508 suggest *P. aeruginosa* biofilm formation correlates with increased available iron levels. Host defenses often actively try to reduce iron levels to hinder bacterial replication, as iron is an important nutrient for bacterial growth. To combat this, *P. aeruginosa* has evolved excess iron receptors and absorption systems (e.g., siderophore and iron chelating molecules).^[^ ^167^ ^]^ Gallium has a similar chemical feature as iron and can be absorbed by bacteria but fails to substitute the function of iron. Therefore, when it is taken up, it inhibits iron‐dependent pathways necessary for bacterial growth and biofilm formation.^[^ ^168^ ^]^ This “Trojan horse” approach has been shown to interfere with the growth and iron metabolism of *P. aeruginosa*, kill phytoplankton in the acute mouse pneumonia model, and reduce the number of bacteria in established biofilms by a factor of 1000 in the chronic biofilm lung infection model.^[^ ^162^ ^]^ Gallium is taken by inhaling. Its absorption by bacteria in vitro is not dependent on the presence of the *P. aeruginosa* siderophore Pyoverdin. In vivo, however, any additional anti‐inflammatory effects that gallium may have on the host beyond direct inhibition of biofilms are not clear.

### 3.4 Targeting Cells in Biofilms

Processes such as biofilm dispersal induction through targeted pathways require the cells to undergo metabolic activity. However, some evidence also suggests that persistent substances residing in biofilms are important in drug resistance.^[^ ^169^ ^]^ Therefore, antimicrobial approaches that consider physical or chemical damage to cells rather than targeting specific cellular processes are attractive. Non‐discriminatory oxidants (e.g., hypochlorite and hydrogen peroxide) have been applied as rinsing agents for wound and endodontic debridement.^[^ ^170^ ^]^ Nevertheless, studies have shown that even strong oxidants like sodium hypochlorite cannot completely remove biofilms likely due to inadequate exposure given the cytotoxicity.^[^ ^171^ ^]^ Wide‐spectrum cationic biguanides like chlorhexidine^[^ ^172^ ^]^ or quaternary ammonium,^[^ ^173^ ^]^ could attach to cell walls and damage membranes. However, these substances penetrate minimally over the time‐ranges used in in vitro dental biofilms, and long‐term exposure is cytotoxic, which makes this strategy unfeasible in clinics

## 4 State‐of‐the‐Art Technologies for Prevention and Treatment of Biofilms

### 4.1 Prevent Biofilm Formation

For a long time, the prevention of biofilm formation has been a hot topic in the research field. The first important role in preventing biofilm formation is to prevent bacteria from attaching to host tissue in order to inhibit infection. This is an enticing therapeutic target for preventing bacterial infection, and multiple approaches have been reported.^[^ ^174^ ^]^ These methods include hindering cell receptors from recognizing adhesive surfaces, inhibiting bacterial adhesion processes, and blocking primary colonizers to hinder the original formation of the biofilm and prevent any future infectious spread by planktonic cells released by the biofilm itself.^[^ ^175^ ^]^

#### 4.1.1 Biofilm Inhibition by Quorum Quenching

Quorum‐sensing (QS) is a system that Gram‐negative and gram‐positive bacteria use to communicate.^[^ ^176^ ^]^ QS modulates the activity of various genes by detecting the concentration of signaling molecules within the bacteria's environment. The signaling molecules in the QS system are denoted as autoinducers.^[^ ^177^ ^]^ QS signaling molecules can be classified into 3 types: N‐acyl homoserine lactones (AHLs)‐based (Gram‐negative bacteria), autoinducing peptide‐based (Gram‐positive bacteria), and autoinducer‐2 (AI‐2)‐based (both Gram‐negative and Gram‐positive bacteria).^[^ ^178^ ^]^ During the biofilm formation, cells secrete QS molecules after initial attachment. These molecules induce changes in gene expression to transform bacteria from a planktonic lifestyle into a sessile form.^[^ ^179^ ^]^ Because QS is so important for the construction of biofilms, it has been proposed that QS inhibition (quorum quenching (QQ)) could be used for the prevention of biofilms.^[^ ^180^ ^]^ Furthermore, QS is important for the expression of many important virulence factors, thus the QS system can be thought of as a promising and novel antimicrobial target.^[^ ^181^ ^]^ The main advantage of using QQ to control biofilms is that it lowers the chance of multidrug resistance. Therefore, this strategy has important clinical significance for the prevention of biofilms.^[^ ^182^ ^]^

According to the size and chemical makeup, QQ agents can be classified into two groups: macromolecular QQ enzymes^[^ ^183^ ^]^ and microparticulate QS inhibitors.^[^ ^184^ ^]^ Enzymatic destruction of AHL molecules is the most noted mechanism of QQ. Many enzymes can catalyze this degradation, but all fall into four distinct classes: lactonases and acylases that hydrolyze the HSL ring and amide bond of AHL, while reductases and oxidases that change the activity of AHL molecules, but do not break down them.^[^ ^185^ ^]^ Another important QQ mechanism is using compounds to antagonize QS inductors; these molecules bind to receptors to prevent the QS signal from being recognized by the cell. Inductor antagonism may be competitive or non‐competitive.^[^ ^186^ ^]^ The last mechanism is inhibiting intracellular signal transduction cascades. It has been demonstrated that savarin, a small molecule inhibitor, disturbs AgrA, a transcriptional regulator of the QS‐involved agr operon, by binding DNA to inhibit the production of RNAIII which contributes to the expression of multiple virulence factors in combination with AgrA.^[^ ^187^ ^]^

#### 4.1.2 Surface Modifications

Implant‐related infections and nosocomial infections not only increase medical costs, but also threaten the health and lives of patients. Treatment of these infections is challenging.^[^ ^188^ ^]^ Despite the best efforts of medical staff to prepare clean areas during surgical implantation, implant‐associated infections continue to persist. Bacterial attachment to the implant is the initial step of the process, succeeded by aggregation of the bacteria to form biofilms that lead to infection. Because of the existence of dense ECM produced by bacteria, the diffusion of antibacterial agents into biofilms is limited, leading to poor therapeutic efficacy and bacterial resistance. Biofilm‐protected bacteria may also be released, leading to new sites of infection.^[^ ^189^ ^]^ It has been shown that eradicating such protected pathogens is nearly impossible. Hence, infected implants must be removed in order to prevent transmission of infection. Considering the consequences and complications of implant‐associated infections, the best approach to care is to prevent infections instead of treating them after they have emerged.^[^ ^190^ ^]^

One potential way to avoid implant‐related infections is to modify the surface of implants with a bacterial‐repellent layer. This layer prevents early bacteria from attaching to the implant surface, which in turn prevents biofilm formation.^[^ ^191^ ^]^ Over the years, a number of surface modification strategies have been reported for producing coatings with anti‐infective activity. At present, anti‐infection modification methods include antifouling material coatings, embedding of antimicrobial therapeutics, and contact‐killing coatings. These methods apply general principles including inhibiting bacterial adhesion to surfaces, destroying bacteria on surfaces, and hindering bacterial growth from the implant surface.

**Figure 6:**
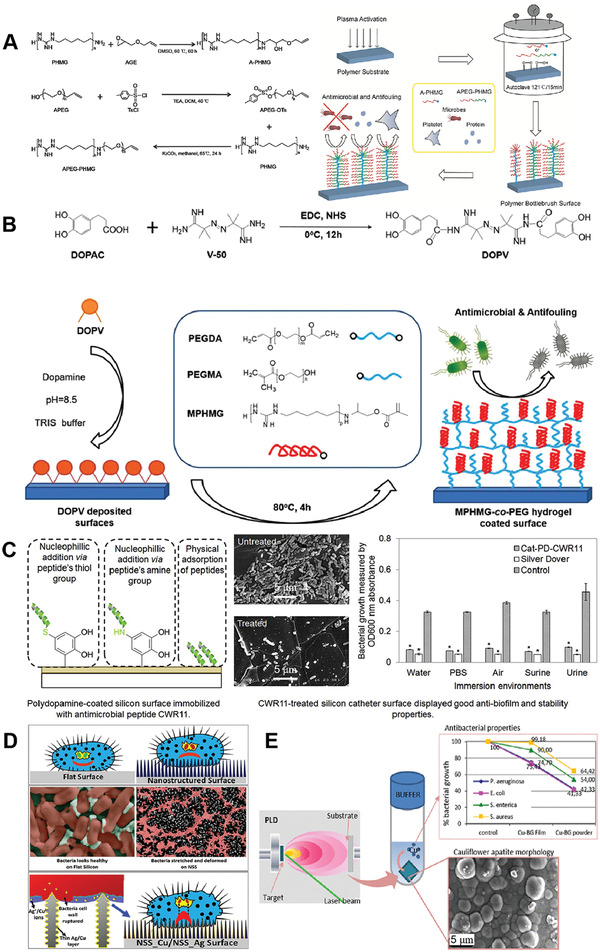
Typical examples of antimicrobial surface coatings. A) Polymer brushes‐based coatings. Reproduced with permission.^[^ ^193^ ^]^ Copyright 2017, Wiley‐VCH. B) Anti‐adhesive hydrogels. Reproduced with permission.^[^ ^194^ ^]^ Copyright 2019, Wiley‐VCH. C) AMP conjugates. Reproduced with permission.^[^ ^197^ ^]^ Copyright 2015, Elsevier. D) Nanopillar array coatings. Reproduced with permission.^[^ ^204^ ^]^ Copyright 2017, American Chemical Society. E) Nanocomposite coatings. Reproduced with permission.^[^ ^206^ ^]^ Copyright 2019, American Chemical Society.

Hydrophilic polymer brush‐based coatings have been a subject of interest; they are resistant to bacterial adhesion due to non‐fouling and protein‐repellent properties. It has been difficult, however, to translate such coatings into medical devices, especially devices made of polymeric materials.^[^ ^192^ ^]^ Su et al. synthesized a block copolymer that combines anti‐infective, antifouling, and surface‐conjugating properties into one molecule and conjugated it to polymeric substrates (**Figure** **6**A).^[^ ^193^ ^]^ The coatings exhibited high antimicrobial efficacy against gram‐positive bacteria, gram‐negative bacteria, and fungi. It demonstrated excellent antifouling activity and was re‐usable for long‐term. Furthermore, the coatings greatly decreased the number of bacteria with 100 000‐fold bacterial reductions in a rodent subcutaneous infection model. Hydrogel coatings have also shown considerable promise, as they have good resistance to protein adsorption and inhibit the biofilm formation of many different bacterial strains. In another work, Su et al. synthesized a novel biomimetic surface‐attachable initiator through the conjugation of 3, 4‐dihydroxyphenylacetic acid and thermal 2, 2′‐azobis (2‐methylpropionamide) dihydrochloride.^[^ ^194^ ^]^ The synthesized initiator can attach to different surfaces like a mussel and initiate surface conjugation (Figure 6B). Hydrogel coatings were synthesized through copolymerization of polyhexamethylene guanidine (antimicrobial) and polyethylene glycol (antifouling) oligomers. In vitro testing of these coatings revealed antimicrobial activity. They were effective against biofilms and had low cytotoxicity. The anti‐microbial activity of these hydrogels was especially efficacious, with a greater than 5‐log reduction in a rodent subcutaneous infection model.

AMPs are another potential solution for the prevention of indwelling device infections and may be an alternative to anti‐microbial coatings. AMPs have broad‐spectrum anti‐microbial activity against bacterial species, fungi, and even viruses. AMPs have been demonstrated to be biocompatible in many assays.^[^ ^195^ ^]^ The titanium surface covalently coated with the major AMP of human cathelicidin LL‐37 is effective against the ESKAPE pathogens (*Enterococcus faecium*, *S. aureus*, *K. pneumoniae*, *A. baumannii*, *P. aeruginosa*, and *Enterobacter* species).^[^ ^196^ ^]^ In a different study, Leong et al. reported a simple and effective method to immobilize a potent synthetic AMP, CWR11, onto catheter‐relevant surfaces.^[^ ^197^ ^]^ Polydopamine (PD) was coated onto a polydimethylsiloxane (PDMS) surface as a thin adherent film to facilitate the attachment of CWR11 onto the PD‐functionalized polymer (Figure 6C). The CWR11‐functionalized PDMS slides showed high levels of antimicrobial and antibiofilm activity. The CWR11‐embedded catheter was potently toxic to bacteria and retained bactericidal for at least 21 days.

Research has reported that the wing surface of insects like the cicada and dragonfly have excellent antibacterial activity.^[^ ^198^ ^]^ Nanopillars on the surface of wings serve as sharp needles that result in holes in the bacterial wall and death of the bacterium. Synthetic replications of these nanopillars with different materials have shown potent bactericidal activity skin to dragonfly and cicada wings.^[^ ^199^, ^200^, ^201^, ^202^, ^203^ ^]^ Sen et al. fabricated sharp‐tipped nanostructures on silicon surfaces (NSS) utilizing the maskless deep reactive ion etching mimicking dragonfly wings (Figure 6D).^[^ ^204^ ^]^ Antimicrobial efficacy of the nanostructured surfaces coated with a thin layer of silver (NSS_Ag) or copper (NSS_Cu) was quantified. NSS_Cu surfaces killed bacteria more efficiently relative to the uncoated NSS. This could be due to the metal ions eluted from coatings and biomimetic nanostructures.

Additional strategy to prevent infections from forming on devices is to incorporate antimicrobial agents directly to kill bacteria. One such strategy is to load device coatings with metallic nanoparticles which release metal ions at a bactericidal concentration to eliminate any bacteria around the surface.^[^ ^205^ ^]^ Boccaccini et al. incorporated 5 wt.% of CuO to 45S5 bioglass materials (Figure 6E).^[^ ^206^ ^]^ This Cu‐doped bioglass served as the substrate for the deposition of bioactive thin films using a pulsed laser. Anti‐microbial analysis of the material revealed it to be more effective in killing Gram‐negative bacteria than killing Gram‐positive bacteria.

It is worth mentioning that some of the surface modification strategies have already been approved by the US Food and Drug Administration (FDA). Most approved therapeutics are related to the hydrophilic coatings on medical devices. According to the FDA website, there are 77 records of approved hydrophilic coating devices as of July 2022. Biocompatibility and durability of hydrophilic coatings show great benefits in the fields of cardiology and urology. Due to the emergence of new coating technologies, the use of hydrophilic coatings in the medical field has increased. With its disposable capacity, devices with hydrophilic coatings are also used in emergency departments and delivery units in medical institutions, while other common devices with hydrophilic coatings are used in the fields of endoscopy and respiratory care.^[^ ^207^ ^]^ There are also 13 records of approved hydrogel‐coated devices, another FDA‐approved surface modification strategy for medical devices. In addition, there are 4 records of approved antimicrobial coated devices.

### 4.2 Eradicate Existing Biofilms

#### 4.2.1 Physical Methods

##### Non‐Thermal (Cold) Plasma

Use of non‐thermal discharge plasmas is one of the physical methods to destroy biofilms. Cold plasma has several distinct advantages compared to traditional sterilization methods because it contains free radicals, charged particles, UV photons, and other reactive substances that are efficient at killing microorganisms.^[^ ^208^ ^]^ Plasma is produced by transferring energy, usually in the form of an electric discharge, into adjacent gas. This energy excites gas particles from their ground state and changes their electron arrangement.^[^ ^209^ ^]^ Plasma is produced most often by the application of an electric field to a neutral gas. When energy is sufficient for both electrons and heavier molecular species, the produced plasma is referred to as “thermal plasma”. Conversely, if the energy level of the electrons is higher than the heavy molecular species, the plasma is known as “cold” or “non‐ thermal”.^[^ ^210^ ^]^ The ability to produce the active agents at ambient or near ambient temperatures (≤ 25–30 °C) and at atmospheric pressure without any vacuum system is a distinct advantage. This is especially pertinent in biomedical applications, because samples cannot be placed inside vacuum chambers. Using plasma for sterilization does not pose any serious health risks for either the operator or the patient and is thus considered safe.^[^ ^211^ ^]^ Besides, the many different reactive species found in plasma likely exert a synergistic effect to increase the efficacy of plasma sterilization. Even at low levels, these reactive compounds have been known to damage or destroy microorganisms. Plasma is generated using air, so using it for sterilization is very cost‐effective.^[^ ^212^ ^]^

**Figure 7:**
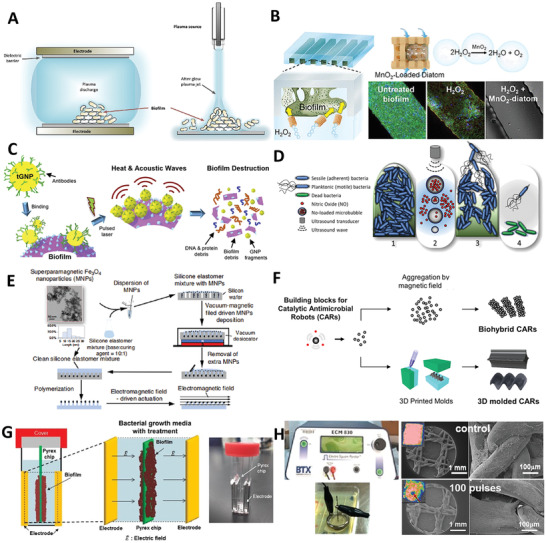
Typical methods of the physical removal of biofilms. A) Non‐thermo plasma. Reproduced with permission.^[^ ^213^ ^]^ Copyright 2018, Elsevier. B) Microbubbles. Reproduced with permission.^[^ ^227^ ^]^ Copyright 2018, American Chemical Society. C) Photothermal therapy. Reproduced with permission.^[^ ^236^ ^]^ Copyright 2019, Elsevier. D) Ultrasound transducer. Reproduced with permission.^[^ ^241^ ^]^ Copyright 2019, John Wiley & Sons Ltd and Society for Applied Microbiology. E) Magnetically driven active topography. Reproduced with permission.^[^ ^247^ ^]^ Copyright 2020, Springer Nature. F) Catalytic antimicrobial robots. Reproduced with permission.^[^ ^257^ ^]^ Copyright 2019, American Association of the Advancement of Science. G) Electrical stimulation. Reproduced with permission.^[^ ^260^ ^]^ Copyright 2015, Springer Nature. H) Pulsed electric fields. Reproduced with permission.^[^ ^263^ ^]^ Copyright 2016, Wiley‐VCH.

A considerable amount of research has investigated the use of non‐thermal plasma (NTP) (e.g., corona discharge, microwave discharges, plasma jet, gliding arc, and dielectric barrier discharge) in the treatment of biofilms (**Figure** **7**A).^[^ ^213^ ^]^ One interesting and notable use of plasma is for the deactivation of oral biofilms. Dental plaque biofilms are made up of a diverse and complicated community of oral microbes; the ability of these communities to cause cavities and gum disease is well known.^[^ ^214^ ^]^ One of the plasma devices for combating these oral biofilms is a plasma needle. It was examined against cariogenic *Streptococcus mutans* in a simulated cavity model, the penetration of the active plasma compounds was measured along with levels of bacterial inactivation.^[^ ^215^ ^]^ Sixty seconds of treatment at 110 and 340 mW power produced a 5–8 mm radius of bacterial inactivation. A different work involved with the use of positive and negative corona discharges on biofilm‐contaminated tooth surfaces to test the efficacy of water electrospraying for decontamination.^[^ ^216^ ^]^ Both types of discharges greatly reduced bacterial concentrations. The magnitude of the reduction correlated with treatment time, the bacterial concentration was reduced by 1–1.3 logs and 2.73 logs at 5 and 10 min. Additionally, water electrospraying via the plasma decreased bacteria by 3.16 orders of magnitude. The treatment was not observed to cause any significant changes to tooth surfaces. In another work, Koban et al. examined the effect of three devices including an atmospheric pressure plasma jet, a hollow dielectric barrier discharge electrode, and a volume dielectric barrier discharge against dental biofilms.^[^ ^217^ ^]^ The analysis revealed the devices reduced *S. mutans* and saliva biofilm levels between 5.38 and 5.67 logs. Chlorhexidine (CHX) was used as a control, showing a log‐reduction of 3.36 and 1.50 on the same biofilms. Additionally, a 5‐log reduction was obtained on *Candida Albicans* biofilms. These results indicated that plasma seems to have higher efficacy than CHX in treating single and multispecies dental biofilms.

NTP could also provide a different method for sterilizing and decontaminating the inner surfaces of medical devices. Implants are vulnerable to infections, and infection is one of the principles which cause implant failure. Therefore, sterilization methods are critical for the success and performance of an implant.^[^ ^218^ ^]^ Ercan et al. assessed the inactivation and inhibition of biofilm formation of *E. coli* and *S. aureus* on UHMWPE, Ti6A14V, 304 SS, and 316L SS surfaces, which are used for acetabular cups and inserts in total knee prostheses.^[^ ^219^ ^]^ Plasma treatment of these discs inactivated 95% of bacterial biofilms after three min of treatment. In a separate study, Matos et al. showed that glow discharge plasma treatment of titanium‐based dental implants increased their wettability.^[^ ^220^ ^]^ The treated surfaces did not show any increase in the proliferation of a multi‐species (*Streptococcus sanguinis*, *Actinomyces naeslundii*, and *Fusobacterium nucleatum*) biofilm. In their continuous study, Matos et al. used the glow discharge plasma method to deposit CHX‐doped thin films on pure titanium (cpTi).^[^ ^221^ ^]^ CHX release was sustained for more than 22 days, leading to a significant suppression of biofilm formation at 48 and 72 h for 50 and 20 min of plasma deposition, respectively.

##### Microbubbles

Microbubbles are micron‐sized bubbles of gas enclosed by a thin layer of lipid or surfactant‐based substance.^[^ ^222^ ^]^ Microbubbles were reported by Charles Joiner for the first time in the late 1960s, who found their existence during an echocardiogram.^[^ ^223^ ^]^ After dozen years, microbubbles were developed as ultrasound contrast agents.^[^ ^224^ ^]^ Fundamentally, microbubbles consist of gases with a high molecular weight stabilized by an outer lipid monolayer. Insoluble gases like fluorinated carbon or sulfur compounds, (e.g., sulfur hexafluoride and decafluorobutane) typically make up the gas core and increase the stability of the microbubble.^[^ ^225^ ^]^

It has been demonstrated that microbubbles can be used to disrupt biofilms. Liu et al. compared the microbubble‐mediated detachment of biofilms from a nylon membrane to chemical cleaning with sodium hypochlorite (NaOCl).^[^ ^226^ ^]^ Roughly 88% of the fixed biomass was detached after 1 h of microbubbling. Contrarily, around 10% of the biomass was detached in the control experiment that did not utilize microbubbling. CLSM images confirmed a complete disruption of EPS, as the images clearly showed an almost total removal of biofilm extracellular polysaccharides and proteins. In another work, Kong et al. hypothesized that engineered microparticles carried by microbubbles could remove a biofilm from a structure by fracturing the EPS and increasing the delivery of antiseptic agents (Figure 7B).^[^ ^227^ ^]^ In order to test this hypothesis, they applied manganese oxide (MnO_2_) nanosheets to a hollow‐cylinder‐shaped diatom biosilica. In an H_2_O_2_ solution, the diatoms doped by MnO_2_ nanosheets, known as the diatom bubbler, released oxygen bubbles and generated self‐motion. Then, the diatoms penetrated the biofilms grown on either flat or microgrooved silicon substrates and kept on producing microbubbles. The microbubbles produced by this process merged, generating enough mechanical energy to break up the biofilm matrix. This allowed H_2_O_2_ molecules to diffuse into the biofilm and kill bacteria.

In addition, microbubbles have demonstrated the ability to improve treatment by increasing a biofilm's susceptibility to drugs. Zhang et al. demonstrated that, when human *β*‐defensin 3 (hBD‐3) was applied together with microbubble destruction, biofilm density was reduced and the number of living cells from two tested *Staphlococcus* strains was significantly decreased when compared with other groups.^[^ ^228^ ^]^ It was also shown that microbubble destruction may boost hBD‐3 activity by hampering the biofilm‐associated gene expression (*icaAD*), decreasing expression of methicillin resistance gene (*MecA*), while also increasing icaR expression. In another work, Wang et al. reported that microbubble destruction damaged the cell wall structure of *S. epidermidis*, which is evidenced by TEM images of floccules and fragments from damaged cells.^[^ ^229^ ^]^ Although the cell membrane was almost intact, the treatment caused an increase in the metabolic activity of bacteria within the biofilm. These changes observed by CLSM and flow cytometry could make them more susceptible to antibiotic killing.

##### Photodynamic and/or Photothermal Therapy

Antimicrobial photodynamic and/or photothermal therapies are promising local strategies for the treatment of antibiotic‐resistant bacterial infections and biofilms.^[^ ^230^, ^231^ ^]^ Over the last few decades, photodynamic therapy (PDT) and photothermal therapy (PTT) have emerged and demonstrated increasing potential in managing bacterial infections partially because of the advances in the synthesis of photosensitizers (PS) /photothermal agents (PTAs). PDT or PTT works by transforming light energy into reactive oxygen species (ROS) or heat, leading to membrane breakup, protein denaturation, and permanent bacterial damage.^[^ ^232^, ^233^ ^]^

Herein, we highlight several notable studies on the use of PDT and/or PTT for biofilm treatment. Some studies described the application of PDT and/or PTT for the treatment of biofilms in vitro. Sayar et al. assessed the efficacy of PDT with toluidine blue (TBO) and indocyanine green (ICG) using 635 nm alongside 808 nm diode lasers in the treatment of *Aggregatibacter actinomycetemcomitans* biofilms grown on Laser‐Lok titanium discs.^[^ ^234^ ^]^ There was a significant difference in the number of bacterial colonies present in the different groups after the intervention. PDT with TBO+635 nm and ICG+808 nm laser treatment significantly reduced bacterial count relative to the control group. TBO alone also significantly reduced the bacterial count as compared with the control group. In a separate study, Bilici et al. combined nanoparticles and organic photosensitizers for efficient removal of planktonic bacteria and their corresponding biofilms.^[^ ^235^ ^]^ In Kirui et al.’ study, the photoinduced antimicrobial activity of ICG, 3‐aminopropylsilane coated superparamagnetic iron oxide nanoparticles (APTMS@SPIONs), and ICG loaded APTMS@SPIONs were tested against planktonic bacteria and biofilms. Successful eradication of biofilms was seen with ICG/laser or ICG‐loaded APTMS@SPION/laser treatment. A dramatic 6.5‐log reduction in CFUs was observed between ICG versus ICG‐loaded APTMS@SPION treatment against *K. pneumoniae* biofilms. They also demonstrated that the gold nanoparticle‐targeted pulsed laser therapy can promote the efficacy of antibiotics in treating MRSA and multidrug‐resistant *P. aeruginosa* biofilms in vitro (Figure 7C).^[^ ^236^ ^]^

Furthermore, many studies have been performed by applying PDT/PTT to remove biofilms in vivo. Yuan et al. presented a phototherapeutic nanoplatform consisting of L‐arginine (L‐Arg), ICG, and mesoporous polydopamine (MPDA), specifically, AI‐MPDA, to combat biofilms.^[^ ^237^ ^]^ Upon NIR irradiation, the biofilm removal was caused by the nitric‐oxide boosted PDT and low‐temperature PTT (≤ 45 °C). This strategy led to severe damage of bacterial membranes. NIR‐irradiated AI‐MPDA nanoparticles stopped bacterial colonization and led to fast healing from contaminated wounds. Notably, the all‐in‐one phototherapeutic platform was nearly 100% effective at eliminating biofilms from an abscess formation model. Cui et al. designed a NIR light‐driven nanoswimmer (HSMV).^[^ ^238^ ^]^ When irradiated by NIR light, HSMV was self‐propelled and penetrated the biofilm within 5 min which was attributed to the photothermal conversion of unevenly distributed AuNPs. The topical thermal energy and heat‐triggered release of vancomycin created a combined chemotherapy and phototherapy system. The self‐propulsion of HSMV increased both the effective distance of PTT and the activity of the antibiotic, leading to a greater than 90% removal of the biofilm. Importantly, HSMV was capable of removing in vivo *S. aureus* biofilms after 10 min of laser irradiation without damaging healthy tissues. PDT/PTT would also be an ideal approach for the elimination of biofilms in biomedical implants. Tan et al. explored the photothermal ability of a red‐phosphorus‐IR780‐arginine‐glycine‐aspartic‐acid‐cysteine coating on titanium bone implants.^[^ ^239^ ^]^ The red phosphorus resulted in the production of singlet oxygen (^1^O_2_) species upon exposure to IR780 which made *S. aureus* biofilms more sensitive to temperature. Biofilms can be destroyed with near‐infrared (808 nm) PTT in vivo without risking damage to normal tissue. Such a method can reduce 96.2% of bacteria in vivo after irradiation at 50 °C for 10 min.

##### Ultrasound

The use of ultrasound for the treatment of biofilms is attractive because it does not use chemicals and is environmentally friendly. Ultrasound is high frequency (>20 kHz) sound waves.^[^ ^240^ ^]^ Ultrasound can produce gas cavities capable of disrupting biofilms or even inactivating microorganisms (Figure 7D).^[^ ^241^ ^]^ On one hand, studies have been conducted on the use of ultrasound in the removal of biofilms in vitro. Granick et al. showed that direct‐contact low‐frequency ultrasound was able to eliminate *S. epidermidis* biofilms grown on the surface of titanium and stainless steel metallic disks. The surfaces of these alloys are similar to those used in surgical implants.^[^ ^242^ ^]^ Crone et al. also demonstrated that a low‐frequency ultrasonic‐assisted wound debridement device was effective in disrupting biofilms in vitro and further enhanced the antibacterial effect of polyhexamethylene biguanide.^[^ ^243^ ^]^ It is worth mentioning that in this study *S. aureus* biofilms were grown in a semi‐solid agar gel consisting of either tryptic soy broth or a wound simulating media, to recapitulate the suspended colonies. Similarly, Torlak and Sert evaluated the effectiveness of ultrasound in combination with benzalkonium chloride in eliminating *Listeria monocytogenes* biofilms on the polystyrene surface.^[^ ^244^ ^]^ The combined treatment resulted in a significantly greater reduction in the number of viable cells in the *L. monocytogenes* biofilms relative to individual treatments. On the other hand, ultrasound is often used in conjunction with other antibacterial agents to treat biofilms in vivo. Yang et al. examined the anti‐fungal synergism produced by combining amphotericin B (AmB)‐loaded poly(lactic‐co‐glycolic acid) (PLGA) nanoparticles (AmB‐NPs) with low‐intensity ultrasound against *Candida albicans* biofilms.^[^ ^245^ ^]^ They showed that the combination treatment of AmB‐NPs with 42 kHz ultrasound produced significant reductions in biofilms when compared to controls, AmB alone, or ultrasound alone. Moreover, similar collaborative effects were observed in a rat subcutaneous catheter biofilm model. The number of CFUs on the catheter reduced significantly after 7 days of continuous therapy with both AmB‐NP and ultrasound, revealing that the biofilm on the catheter surface was severely destroyed. In another study, to prevent spinal fusion infection, Delaney et al. designed a polyether ether ketone (PEEK) antibiotic reservoir clipped onto the metal fixation rod and achieved sustained release of antibiotics for more than 7 days, succeeded by a bolus release due to the rupture of the reservoir membrane by ultrasound.^[^ ^246^ ^]^ In human fluids, higher levels of vancomycin were needed to achieve a substantial reduction in adherent bacteria when compared to common bacterial mediums. In order to obtain such levels of released vancomycin, polylactic acid was coated to the porous PEEK puck to achieve both slow and ultrasound‐controlled release, which were enough to block *S. aureus* adhesion to implants. The design was additionally revised to a one‐/two‐hole cylindrical PEEK depot that was able to attach to a spinal rod for clinical applications.

##### Magnetic Manipulation Systems

Control of the movement of micron‐scale objects by the magnetic field can generate sufficient forces that may be used to prevent bacteria from attaching to a surface and to remove established biofilms on demand. For example, Gu et al. demonstrated adjustable dynamic surface topographies composed of micropillars capable of beating at controlled frequency and magnitude in an electromagnetic field (Figure 7E).^[^ ^247^ ^]^ The optimized active topographies could reduce the load of mature biofilms of uropathogenic *E. coli* (UPEC), *P. aeruginosa*, and *S. aureus* by 3.7 logs. Additionally, the disengaged bacteria were sensitized to bactericidal antibiotics at a level similar to the planktonic cells. According to the same principle, they prepared catheters with programmable surface topographies that can keep clean for more than one month under the flow of simulated urine solution, but the catheters without such topographies were occluded by UPEC biofilms for less than 5 days. In another study, Elbourne et al. showed that liquid metal droplets made of gallium were magnetic responsive and capable of converting their shapes to develop sharp edges upon exposure to a low‐intensity rotating magnetic field for 90 min, resulting in physical destruction of bacterial cells and disruption of the compact EPS of biofilms.^[^ ^248^ ^]^

Magnetic manipulation has also been used for the removal of biofilms in vivo. Yang et al. examined the efficacy of superparamagnetic iron oxide nanoparticles and amoxicillin co‐loaded chitosan/poly (acrylic acid) nanoparticles in treating *Helicobacter pylori* biofilms.^[^ ^249^ ^]^ They demonstrated that the nanoparticles may inhibit the biofilm and exert the bacteria‐killing effect against *H. pylori* due to amoxicillin. Besides, the nanoparticles can stick to the gastric mucosa due to chitosan's mucoadhesive property and quickly penetrate the mucosal membrane when exposed to a magnetic field, which can prolong the time drugs remain in the gastry and decrease the dose and medication time, resulting in effective removal of *H. pylori* biofilms. In a different study, Quan et al. used iron oxide nanoparticles (MIONPs) to interact with staphylococcal pathogens through magnetic channel digging followed by gentamicin treatment to eradicate biofilms.^[^ ^250^ ^]^ It was demonstrated that MIONPs can promote the efficacy of gentamicin around 10 times against *S. aureus* Xen36 biofilms in a mouse subcutaneous infection model. Moreover, the highest CFU decrease was attributed to the MIONPs’ magnetic movement across the biofilms. At the same time, the isolated tissue around the infection location contained fewer inflammatory cells. Additionally, magnetic materials have also been applied to assist in addressing the problem of implant‐associated infections. Wang et al. attempted to destruct biofilms in implant‐associated infections using a magneto‐based cooperative treatment.^[^ ^251^ ^]^ It was shown that CoFe_2_O_4_@MnFe_2_O_4_ nanoparticles (MNPs) can break up compact biofilms due to the hyperthermia generated in an oscillating magnetic field. Nitrosothiol‐coated MNPs (MNP‐SNOs) were able to pass through the channels in dispersed biofilms to eliminate bacteria because of the combinatorial effect including the released NO from nitrosothiols and the magnetic hyperthermia. Furthermore, MNP‐SNOs can provoke macrophage‐related innate immunomodulation to avoid the recurrence of implant‐related infections. Meanwhile, the evident antimicrobial effect of this platform is further validated in a rat implant‐related infection model.

##### Catalytic Therapy

Various catalytic materials have been used for the treatment of biofilms via the generation of ROS. ROS are critical for the immune system to combat microorganisms. ROS can be produced by the NADPH oxidase in the innate immune cells.^[^ ^252^ ^]^ ROS is capable of deactivating bacteria by destructing the proteins, DNA, and polysaccharides in an irreversible way. Significantly, ROS can break down the structure of mature biofilms.^[^ ^253^ ^]^ Among these catalytic materials, nanozyme and polyzyme, which may promote the catalytic processes of natural enzymes and control the redox level of cells, in particular on ROS, have been regarded as promising agents to kill bacteria and eliminate biofilm.^[^ ^254^ ^]^ Huang et al. reported a catalytic polyzyme consisting of lipophilic transition metal catalysts loaded with self‐assembled polymer nanoparticles.^[^ ^255^ ^]^ These nanoparticles provided a protective environment for the catalysts. The polyzyme was demonstrated to be able to diffuse into biofilms and get rid of embedded bacteria via the bioorthogonal activation of a pro‐antibiotic. In another study, Liang et al. investigated carbon dots (CDs)@platinum nanoparticles (PtNPs) (CPP) nanoflare by integrating the CDs with PtNPs a peroxidase‐mimicking nanozyme for antibacterial/antibiofilm applications.^[^ ^256^ ^]^ The CPP catalyzed H_2_O_2_ to generate ROS, exhibiting improved decontamination of pathogens. Importantly, the CPP nanozyme demonstrated major biofilm removal and wound healing efficacy in vivo due to endogenous H_2_O_2_ in the acidic infection tissues. Such a strategy may avoid antibiotic resistance to a great extent.

In recent years, many new catalytic materials have been applied to remove biofilms in vivo. In a study, to address both drug and mechanical resistance problems, Hwang et al. demonstrated catalytic antimicrobial robots (CARs) capable of killing bacteria and disrupting the EPS through catalytic activity and simultaneously removing the biofilm from surfaces based on their magnetic actuation property (Figure 7F).^[^ ^257^ ^]^ In their studies, two small‐scale robotic platforms with the incorporation of iron oxide nanoparticles serving as both magnetic actuator and enzyme‐like (e.g., peroxidase) catalysis were shown to destroy and remove biofilms in inaccessible, narrow spaces, or on surfaces. Robots were controlled by the magnetic field and moved along certain directions to specifically wipe off dead bacteria and biofilm remains. Their study demonstrated the capability of precisely targeting biofilm eradication in unreachable sites or on various surfaces. In another study, Long et al. developed atomic‐catalytic centered, hedgehog‐like particles on a micron scale for combatting MRSA through recapitulating the “capture and killing” feature of macrophages.^[^ ^258^ ^]^ The capture and killing effect of synthesized particles was attributed to the hedgehog topography and catalytic production of ROS (e.g., •O_2_^−^ and HClO with Fe_2_N_6_O as catalytic centers). These macrophage mimicking particles showed a low MIC against MRSA and enhanced wound healing in a rabbit skin wound infection model. It seems that the use of physical removal by magnetic actuation and ROS killing rendered by catalytic reactions may represent an efficient combinatorial approach to eradicate biofilms.

##### Electrical Stimulation

Electrical stimulation has been used in several clinical scenarios including stimulation of nerves and muscles, promotion of wound healing and bone regeneration, and electrophoretic drug delivery.^[^ ^259^ ^]^ Electrical stimulation has also been studied for the treatment of biofilms and it was first proved to be effective in treating biofilms in vitro. Kim et al. showed the combination of electrical energy applied through alternating (AC), direct (DC), and superimposed (SP) potentials and antibiotics to treat *E. coli* biofilms (Figure 7G).^[^ ^260^ ^]^ They found that the energy rather than the type of electrical signal (e.g., AC, DC, or SP) mainly contributes to the antibiofilm efficacy. The results from this study shed new light on how electrical stimulation works and open opportunities to incorporate the bioelectric effect into the management of biofilms in clinics.

Electrical stimulation has also been demonstrated to treat contaminated medical devices and biomaterial‐associated infections. Ercan et al. showed the reduction of *S. aureus* growth on traditional titanium because of anodization and electrical stimulation.^[^ ^261^ ^]^ The results showed a dramatic decline in the *S. aureus* biofilm formation after culture for 2 days upon electrical stimulation coupled with anodized nanotubular titanium, relative to non‐anodized titanium without stimulation, which was attributed to the fluorine existing on the exterior of anodized tubular titanium. In another study, Ehrensberger et al. examined the combination of electrical stimulation of titanium with cathodic voltage and the use of antibiotics (e.g., vancomycin and gentamicin) to avert bacteria attachment and periprosthetic joint infection.^[^ ^262^ ^]^ It was demonstrated that electrical stimulation alone or in combination with antibiotics can fully get rid of MRSA and *P. aeruginosa* biofilms. Synthetic mesh is the most common repair material used for reinforcement of ventral hernias, and infection of implanted mesh could lead to significant morbidity for patients. In view of this, Khan et al. examined the influence of pulsed electric fields on *P. aeruginosa* biofilms within polypropylene meshes (Figure 7H).^[^ ^263^ ^]^ They studied how the electric field strength and the number of pulses affected the removal of bacteria. Their data suggested that the enhanced treatment efficiency was mainly attributed to the increased number of applied pulses. Moreover, the bacterial killing rate was illustrated as a function of the electrical field which was able to fit the statistical Weibull model for 150 and 300 pulses.

Electrical stimulation could offer an alternative treatment method for biofilms in skin wounds. Ashrafi et al. investigated the efficacy of electrical stimulation against biofilms in biofilm‐containing human skin wound models and simultaneously monitored the response of treatment in a non‐invasive manner using volatile organic compound (VOC) biomarkers.^[^ ^264^ ^]^ It was shown that electrical stimulation can significantly reduce the viability, metabolic activity, and biomass of MSSA and *P. aeruginosa* in vitro and ex vivo relative to no treatment. It is worth noting that the profiles of VOC produced by bacteria varied significantly after electrical stimulation which could be used as biomarkers for diagnosis and monitoring of wound infections responded to different treatments. The above‐mentioned physical methods for biofilm eradication may allow the development of different intervention strategies to combat biofilms. We further summarize the advantages and shortcomings of different physical methods for eradicating biofilms in **Table** **1**.

**Table 1:** The advantages and shortcomings of different physical methods for eradicating biofilms

Physical methods	Advantages	Shortcomings	Refs.
Non‐thermal (cold) plasma	*Antimicrobials generated locally *High level of oxidation/reactive species renders resistance unlikely	*Accessibility of plasma *Biofilm EPS may protect cells deeper down *Response to plasma is species‐dependent *Highly localized	[265]
Microbubbles	*Physical action reduces probability of resistance *Readily combined with irrigants and shear	*Accessibility *Biofilm can resist removal due to the viscoelasticity *Residual cells may remain	[266]
Photodynamic and/or photothermal therapy	*Antimicrobial activity can be controlled locally *Can be readily combined with surface modifications	*Delivery to infected site and transport into biofilm *Accessibility of light *Biofilm EPS may protect cells * Cytotoxic effects due to the generated heat	[265]
Ultrasound	*Readily projected through skin and soft tissue *Local delivery *Physical action reduces probability of resistance	*Limited targeting *Influence of pressure waves on viscoelastic biofilms not well understood *Local delivery limited to small and accessible areas	[267]
Magnetic manipulation systems	*Avoiding the limited tissue penetration *Physical targeting *Efficiently penetrate biofilm *Biofilm mechanical destruction	*Difficult to target in vivo	[247, 268]
Catalytic therapy	*Use via the generation of reactive oxygen species (ROS), deactivating bacteria by destructing the proteins, DNA, and polysaccharides *Damage the EPS of biofilms and weaken the resistance of bacteria in biofilms to environmental stress	*Low therapeutic efficacy without the addition of H_2_O_2_ *Lack of targeting ability to bacterial biofilms	[257, 269]
Electrical stimulation	*Projected through induction or connected wires *On‐demand antimicrobial generation *Also promote wound healing	*Electrochemistry of body fluids not well understood * Heat generation *Delivery of electrical currents to deep tissue *Cytotoxicity	[270]

#### 4.2.2 Chemical Methods

##### Bacteriophages

Bacteriophages are one of the most numerous and diverse groups of viruses. Bacteriophages contaminate bacteria, take control over their machinery, reproduce intracellularly, and are secreted by host cell lysis.^[^ ^271^ ^]^ Being antibiofilm therapeutics they are superior to antibiotics due to their defined, benign, self‐reproducing, and self‐restraint characteristics.^[^ ^272^ ^]^ Phage‐borne depolymerases break down the biofilm EPS the physical hurdle for antibiotic diffusion and lead to substantial biofilm destruction. Recently, bacteriophages have been frequently used in the treatment of biofilms in vitro. Papadopoulou et al. examined the efficacy of bacteriophages in combating *Flavobacterium psychrophilum* biofilms in vitro in terms of inhibiting the formation and reducing the biomass of biofilms.^[^ ^273^ ^]^ Their results indicated lytic bacteriophages could effectively prevent the formation of *F. psychrophilum* biofilms, and simultaneously decrease the total mass of established biofilms. Moreover, combining different phages (e.g., Fpv‐9 and Fpv‐10) could result in a significant decrease in the total mass of formed *F. psychrophilum* biofilms. In another study, Wroe et al. developed a hydrogel that was able to load *P. aeruginosa* bacteriophage and deliver active phage to the infected area through injection.^[^ ^274^ ^]^ The bacteriophage‐loaded hydrogels can efficiently eliminate the host bacteria with different phenotypes (e.g., planktonic and biofilm) exerting no effects on the metabolism of human mesenchymal stromal cells. The murine segmental bone defects with *P. aeruginosa* infection were treated with the bacteriophage‐loaded hydrogels, resulting in 4.7 times CFU count decrease in the infected area as opposed to hydrogels without incorporation of bacteriophage after implantation for 7 days.

Treating biofilms with multiple types of antimicrobials with different acting mechanisms could be more efficacious to combat biofilms composed of distinct bacterial strains when compared to the treatment with a single antimicrobial agent.^[^ ^275^ ^]^ Plota et al. investigated the efficacy of daptomycin and bacteriophage K in killing *S. aureus* and *S. epidermidis* strains.^[^ ^276^ ^]^ They noticed there were no dramatic disparities between species. However, daptomycin showed higher efficacy when using medium and high concentrations of the drug. Bacteriophage K exhibited higher killing efficacy against more susceptible strains. When administering both daptomycin and bacteriophage K with high concentrations, they can obtain the best in vitro antibiofilm effect. The activity of bacteriophages has also been demonstrated against clinically‐relevant bacteria in vivo including antibiotic‐resistant strains. Their utility may be especially impactful in clinical settings where antibiotics fail. For example, Wang et al. assessed the efficacy of a bacteriophage ɸWL‐3 together with antibiotics in the treatment of an antibiotic‐resistant *E. coli* biofilm.^[^ ^277^ ^]^ The combinatorial treatment enhanced the antibiofilm efficacy of antibiotics, in particular after sequential administrations, leading to the reduction of 51‐fold of the minimal biofilm bactericidal concentration. Co‐administration of ɸWL‐3 and fosfomycin showed better survivability relative to monotherapy in a *Galleria mellonella* invertebrate *E coli* ATCC 25 922 infection model.

##### Nanomaterials

Nanomaterials can offer antimicrobial properties and have been used as a carrier for antibiotics with great stability and good biocompatibility. Nanomaterials with a high surface‐area‐to‐volume ratio have a large contact surface with the bacterial cell membrane, exhibiting high therapeutic efficacy in treating biofilms.^[^ ^278^ ^]^ It is believed that the use of nanomaterials for the development of antibiofilm approaches is promising in combating bacterial infections and resistance. Three steps are normally involved when nanomaterials interact with biofilms. The first is to deliver nanomaterials to the surrounding area of biofilms. The second is to adhere to the exterior of biofilms. The third is to infiltrate into biofilms. The accomplishment of each step is controlled by numerous aspects such as physicochemical properties of nanomaterials, biofilm's EPS, and surroundings.^[^ ^279^ ^]^

##### Metal (Oxide) Nanoparticles

Metal nanoparticles, metal oxide nanoparticles, or their combinations have been used to overcome limitations that occur in traditional antibiotics such as hindrance in penetration and excretion from systems after therapy.^[^ ^289^ ^]^ Many studies have demonstrated metal nanoparticles are capable of providing antimicrobial or antibiofilm effects. The mechanisms involved in the bactericidal activity of metal nanoparticles include the generation of ROS, release of ions, and influence on the cell membrane of bacteria.^[^ ^282^ ^]^ Among the emerging metal nanomaterials, much attention has been paid to silver nanoparticles (AgNPs) due to their potent antimicrobial activities and increasing applications in many nanomedicine fields.^[^ ^290^ ^]^ Gurunathan et al. investigated the efficacy of silver nanoparticles in conjunction with traditional antibiotics in treating different human pathogenic bacteria and their corresponding biofilms.^[^ ^291^ ^]^ Combining sublethal concentrations of antibiotics and A. cobbe‐mediated synthesized AgNPs showed a dramatic increase in ROS production and cell death compared to AgNPs or antibiotics only. When co‐incubation of AgNPs, the antibacterial efficacy of tested antibiotics, in particular ampicillin and vancomycin, was greatly enhanced in combating Gram‐negative and Gram‐positive bacteria. Recently, AgNPs were also combined with other materials to exert or enhance their antibiofilm effect. Guo et al. developed silver nanocomposites by reducing AgNO_3_ in the presence of a carbohydrate polymer compatible with a living system and a cationic polymer capable of the destructing cell membrane.^[^ ^292^ ^]^ The nanocomposites were demonstrated to effectively break up and get rid of the established biofilms. Furthermore, the nanocomposites can eliminate the biofilms established on the silicone grafts implanted. Such nanocomposites could represent a novel approach to removing biofilms on built‐in medical implants.

Besides metal NPs, NPs made of metal oxides, such as TiO_2_, Fe_3_O_4_, ZnO, CuO, MgO, Al_2_O_3_, etc., and their nanocomposites were also widely used as antibiofilm agents. Metal oxide NPs have been demonstrated to interact with bacteria based on electrostatic interactions, resulting in alterations of the bacterial cell wall, enzyme, or DNA pathways via generated ROS.^[^ ^293^ ^]^ Recently, Naseer et al. prepared CuO NPs from Cassia fistula and Melia azedarach leaf extracts using Cu(NO_3_)_2_.^[^ ^294^ ^]^ The resultant NPs can inhibit 92.5% and 99.5% of *K. pneumonia* and *H. pylori* biofilms, respectively, by disrupting bacterial cell shape and damaging DNA. Similarly, metal oxide NPs could work together with other materials to exert greater antimicrobial activities. Banerjee et al. showed zinc oxide NPs with pancreatin (PK) enzyme (ZnONPs‐PK) were able to better remove MRSA biofilms than ZnO NPs or PK alone,^[^ ^295^ ^]^ indicating it was promising to use ZnONPs‐PK for treating infected swine dermis.

Use of different combinations of metal nanomaterials could have a synergistic antibiofilm effect. For example, Jang et al. prepared Ag and Cu bimetallic NPs on a graphene oxide surface.^[^ ^296^ ^]^ The bimetallic NPs were able to eradicate the *P. aeruginosa* biofilm which grew in a microchannel. In addition, the local administration of bimetallic NPs could result in fast and efficient healing of a mouse biofilm‐infected skin wound model, suggesting their potential applications in combating skin or wound infections caused by antibiotic‐resistant bacteria. In another study, Padilla‐Cruz et al. developed Ag‐Fe bimetallic NPs by reduction from a chemical isolated from the leaves of *Gardenia jasminoides*.^[^ ^297^ ^]^ The synthesized NPs showed a synergistic killing effect against multidrug‐resistant bacteria due to the two different metal components. The potential antimicrobial mechanism for bimetallic NPs could be due to the oxidation of the thiol side chain in cysteine existing in cell wall proteins by Fe^+^ causing changes in penetrability and oxidative stress induced by the released Ag^+^ in the cytoplasm contributing to DNA damage and membrane destruction. Other than antimicrobial activity, the bimetallic nanoparticles were also responsive to an external magnetic field because of the iron component.

##### Carbon‐Based Nanomaterials

Carbon‐based nanomaterials (CNMs) are broadly applied in many areas including antibiofilm agents because of their unique mechanical features, thermal persistence, and chemical stability.^[^ ^298^ ^]^ The potential antimicrobial mechanisms for CNMs could include physical and mechanical disruption, oxidative stress induction, photothermal reaction, photocatalytic reaction, lipid extraction, bacterial metabolism inhibition, segregation by encasing, and cooperative effects.^[^ ^299^ ^]^ CNMs have been applied to eradicate the formed biofilm in vitro. Li et al. designed a nanosystem based on carbon dots stemming from the ashes of sintered L‐lysine powder (CD_Lys_) and modified by a pH‐responsive copolymer.^[^ ^300^ ^]^ The nanostructures could quickly penetrate into the well‐established *S. aureus* biofilm due to their hydrophilic corona and then break up into two portions including ‐NH_2_ ended copolymer and CD_Lys_ due to the responsiveness to the low‐pH environment. The copolymer exhibited antimicrobial activity due to the coulomb interaction between negatively charged bacteria surfaces and the protonated ‐NH_2_ groups. The released CD_Lys_ were distributed across the compact biofilm, producing ROS to kill bacteria. Meanwhile, CNMs widely used as antibiofilm agents to eradicate the biofilm in vivo sometimes work cooperatively with other materials. Fan et al. demonstrated metal‐organic‐framework (MOF)‐derived 2D carbon nanosheets (2D‐CNs) capable of eradicating bacteria topically.^[^ ^301^ ^]^ The MOF‐derived, ZnO‐doped carbon on graphene (ZnO@G) showed prolonged Zn^2+^ release, efficient ions infiltration, and effective photothermal killing synergistically enhancing the destruction of bacterial membranes and intracellular substances. The 2D‐CNs capable of eradicating biofilms were also able to effectively treat skin wound infection via photothermal effect. In another study, Wang et al. developed nitrogen‐doped carbon quantum dots (N‐CQDs) which were effective to kill MRSA without generating resistance, inhibit the biofilm formation, and remove established biofilms.^[^ ^302^ ^]^ It was also shown that the administration with N‐CQDs was able to greatly decrease the bacteria CFU count in the infected tissue and enhance wound closure in a mouse infectious skin wound model.

##### Polymer‐Based Nanoparticles

Polymeric NPs have many unique features for antimicrobial agent delivery and have been demonstrated as a powerful tool in combating biofilms.^[^ ^303^ ^]^ The polymers including poly (lactic‐co‐glycolic acid) (PLGA), poly(*ε*‐caprolactone) (PCL), and chitosan (CS) are widely applied for preparing NPs in biofilm treatment. PLGA nanoparticle platforms lead the way for the development of multifunctional drug delivery systems because of the easy surface modification, cell engulfment, and desired release profiles.^[^ ^304^ ^]^ Notably, Iannitelli et al. encapsulated carvacrol in PLGA nanocapsules to obtain a suitable drug delivery system that could treat biofilm‐associated infections.^[^ ^305^ ^]^ In another study, Hasan et al. designed a polyethyleneimine/diazeniumdiolate‐doped PLGA NPs which could attach to the EPS of MRSA biofilm, leading to enhanced antibiofilm activity.^[^ ^306^ ^]^ The treatment with these NPs showed complete biofilm dispersal, reduction in bacterial burden, and acceleration in wound healing in a diabetic mouse MRSA biofilm‐infected wound model. In addition, Deepika et al. reported PEG‐PLGA NPs co‐loaded with natural and synthetic drugs (e.g., rutin and benzamide) combating the biofilms formed by MDR strains of *S. aureus* and *P. aeruginosa*.^[^ ^307^ ^]^ Such NPs can inhibit the adherence and attachment of bacteria to the substrate because of their interference with biofilm formation via a non‐growth suppressive process.

CS is the biocompatible, biodegradable, and non‐toxic N‐deacetylated derivative of chitin and has been broadly employed in pharmaceutical and medical industries.^[^ ^308^ ^]^ In addition, it has been reported that chitosan and its derivatives show antibiofilm activities.^[^ ^309^ ^]^ CS NPs may serve as an ideal carrier for drug delivery in the eradication of biofilms.^[^ ^310^ ^]^ Tan et al. demonstrated oxacillin and Deoxyribonuclease I (DNase) co‐loaded CS NPs have higher activity against *S. aureus* biofilms in vitro than oxacillin‐loaded NPs and free drugs.^[^ ^311^ ^]^ Such NPs could prohibit the formation of biofilms and disrupt the established biofilms effectively via degrading eDNA and decreasing the number of survived cells and the thickness of biofilms. In another study, Shrestha et al. developed CS NPs functionalized with rose‐bengal to enhance antibiofilm effects, in which the antimicrobial activity was attributed to the adherence to the bacterial cell surface, penetration into the membrane, and lysis of the cells.^[^ ^312^ ^]^ These NPs were demonstrated to decrease the viability of *Enterococcus faecalis* and disrupt the biofilm structure. In addition, Panwar et al. prepared ferulic acid‐loaded CS NPs for combating *Candida albicans* (*C. albicans*).^[^ ^313^ ^]^ Their results revealed that drug‐loaded NPs disrupted the structure of *C. albicans* biofilms and decreased about 22.5% of metabolic activity of *C. albicans* relative to free drugs and blank NPs.

Although PCL is not used so frequently as PLGA and CS, it has been applied together with other materials (e.g., polymers and lipids) for the delivery of antimicrobial agents. Cheow et al. investigated levofloxacin‐loaded PCL NPs against *E. coli* biofilms, revealing their critical role in inhaled therapy against biofilm infections, where the rapid release of levofloxacin in the early stage allows a high initial drug concentration and the subsequent prolonged release allows a high‐sustained drug concentration to effectively prevent biofilm growth and reduce the exacerbation.^[^ ^314^ ^]^ Kho et al. also explored respirable antibiotic‐loaded PCL NPs which are effective in eradicating 99.9% of the *E. coli* biofilm.^[^ ^315^ ^]^ In a different study, Mir et al. prepared carvacrol‐PCL NPs with great potential against biofilms composed of multidrug‐resistant pathogens.^[^ ^316^ ^]^

**Figure 8:**
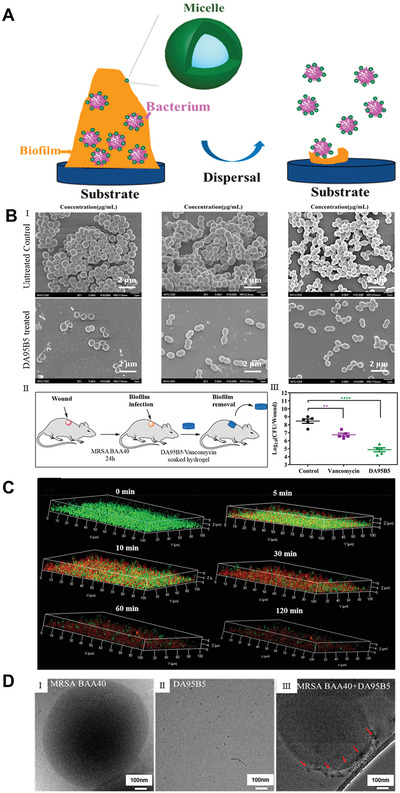
Block copolymer nanoparticles remove biofilms via nanoscale bacterial debridement. A) The possible mechanism of preformed biofilm removal by DA95B5 NPs. B) I): Representative SEM images of Gram‐positive bacteria. MRSA BAA40, VRE, and OG1RF biofilms on pegs of the MBEC biofilm inoculator before and after DA95B5 treatment (with 128 µg mL^−1^). II): Schematic illustrating DA95B5/vancomycin‐soaked hydrogels against MRSA BAA40 biofilms in an established murine excision wound model. III): Log CFU per wound from hydrogels alone and DA95B5‐soaked (2.5 mg kg^−1^) and vancomycin‐soaked (2.5 mg kg^−1^) hydrogels. Each type of hydrogel was applied 3 times at 4 h intervals before plating for CFU determination on agar plates. C) Penetration profiles of polymers at different time points. Time‐lapse 3D confocal images of MRSA BAA40 biofilms treated by DA95B5 at 128 µg mL^−1^ with incubation times of 0, 5, 10, 30, 60, and 120 min, showing the dispersal of biofilms (Green is live bacterial cells, Red is dead bacterial cells). D) Effect of DA95B5 on the properties of three Gram‐positive strains. Cryo‐TEM images showing I) MRSA BAA40 bacteria, II) DA95B5 NPs in PBS buffer, and III) the location of DA95B5 NPs in the MRSA BAA40 bacteria. The arrows denote NPs coated onto the bacterial surface. Reproduced with permission.^[^ ^317^ ^]^ Copyright 2018, American Chemical Society.

In addition to these three most common polymer‐based NPs mentioned above, there are also many studies on multi‐block and multi‐functional polymeric NPs to eradicate biofilms in vivo. Li et al. demonstrated the self‐assembly of block copolymer DA95B5, dextran‐block‐poly ((3‐acrylamidopropyl) trimethylammonium chloride‐co‐butyl methacrylate) into NPs with a non‐fouling dextran shell and a cationic core which can effectively remove established biofilms of different multidrug‐resistant bacteria.^[^ ^317^ ^]^ These NPs penetrated into biofilms and adhered to bacteria without killing them, rather, they facilitated the slow disperse of bacteria, probably due to the enhanced solubility of the bacteria‐nanoparticle complex by the dextran shell (**Figure** **8**A). The field‐emission scanning electron microscopy (FESEM) images showed that the cell number of gram‐positive bacteria in the biofilm decreased after administration with one dose (128 µg mL^−1^) of DA95B5 (Figure 8B (I)). DA95B5 was used as a solution in combination with a hydrogel dressing, exhibiting better efficacy (a 3.6‐log reduction of MRSA) than vancomycin in a murine excisional biofilm‐infected wound model (Figure 8B (II)). CLSM was used to confirm the diffusion of DA95B5 labeled with rhodamine into MRSA BAA40 biofilms through a time‐lapse imaging. As indicated in Figure 8C, DA95B5 can diffuse into the MRSA BAA40 biofilm in less than 5 min, likely due to attractive interactions between charged molecules. Longer time‐lapse imaging shows a significant drop in green fluorescent signals indicated by labeled MRSA BAA40 bacteria, suggesting DA95B5 can disperse the MRSA BAA40 biofilm. The interaction of DA95B5 NPs with MRSA BAA40 bacteria was also investigated by cryo‐TEM (Figure 8D), showing the accumulation of DA95B5 near the surface of bacteria as NPs without breaking up the intact cell membrane. These observations show that DA95B5 NPs can attach to the surface of Gram‐positive bacteria without diffusing into the cytoplasm, indicating their non‐bactericidal characteristics. Administration of DA95B5 NPs seems to be a new strategy for biofilm eradication through the bacterial debridement on nanoscale unlike traditional antibiotic therapy potentially developing drug resistance in bacteria and nonresponsive to multi‐drug resistant bacteria. Such a method should be as effective against resistant bacterial strains as against sensitive strains, indicating many potential applications.

##### Lipid‐Based Nanoparticles

Lipid‐based NPs are ideal carriers for drug delivery to biofilms. Three of the most common categories include solid lipid NPs (SLN), nanoemulsions, and liposomes which have been reported for delivering antibacterial/antibiofilm agents. These carriers are critical for the elimination of bacteria.^[^ ^318^ ^]^

SLN has the potential to deliver antimicrobial agents for a long duration, while reducing the negative effects of drugs due to the prevention of the environment from directly contacting the drugs.^[^ ^319^ ^]^ SLN shows good stability as antimicrobial agent delivery systems in vivo and is superior to traditional drug formulations. Singh et al. developed SLNs of cefuroxime axetil against *S. aureus* biofilm, showing twofold higher antibiofilm activity than free drug in vitro.^[^ ^320^ ^]^ In a different study, Anjum et al. prepared anacardic acid‐loaded SLNs, and then coated with chitosan and DNase using a layer‐by‐layer approach due to electrostatic interactions.^[^ ^321^ ^]^ The design rationale for coating DNase and chitosan was based on the hypothesis that DNase can disrupt the biofilm by degrading the e‐DNA and chitosan can enhance the adherence to biofilms due to its positive charge. The synthesized SLNs showed lower MBIC and minimum biofilm eradication concentration against *S. aureus* biofilms when compared with control, demonstrating the superior design of anacardic acid formulations. In another study, Akhtari et al. fabricated rifampin and cis‐2‐decenoic acid co‐loaded SLNs with higher antibiofilm efficacy in vitro than free drugs in particular in the biofilm‐forming phase.^[^ ^322^ ^]^

Nanoemulsions with good kinetic stability greatly improve the solubility of antimicrobial agents. Notably, the utilization of these nanoemulsions could promote antimicrobial efficacy.^[^ ^323^ ^]^ Song et al. investigated the antibacterial and antibiofilm efficacy of designed chlorhexidine acetate nanoemulsions.^[^ ^324^ ^]^ The nanoemulsions had higher efficacy in combating MRSA in comparison to its water solution. It also showed higher efficacy in the prevention of biofilm formation and eradication of the biofilm. In another study, Quatrin et al. successfully produced nanoemulsions that contain *Eucalyptus globulus* oil.^[^ ^325^ ^]^ The nanoemulsions with a size of around 76 nm and a zeta potential of −9.42 mV showed antimicrobial and antibiofilm activities against three tested species of Candida but without exhibiting antimicrobial activity against *P. aeruginosa* likely because of the low oil content. Moreover, Fang et al. examined the antimicrobial and antibiofilm efficacy of cetylpyridinium chloride‐incorporated nanoemulsions against MRSA.^[^ ^326^ ^]^ They found the smaller the droplet size, the higher the antibiofilm effect of nanoemulsions. The nanoemulsions with small size exhibited excellent antibiofilm activity, resulting in a tenfold CFU decrease relative to the control.

**Figure 9:**
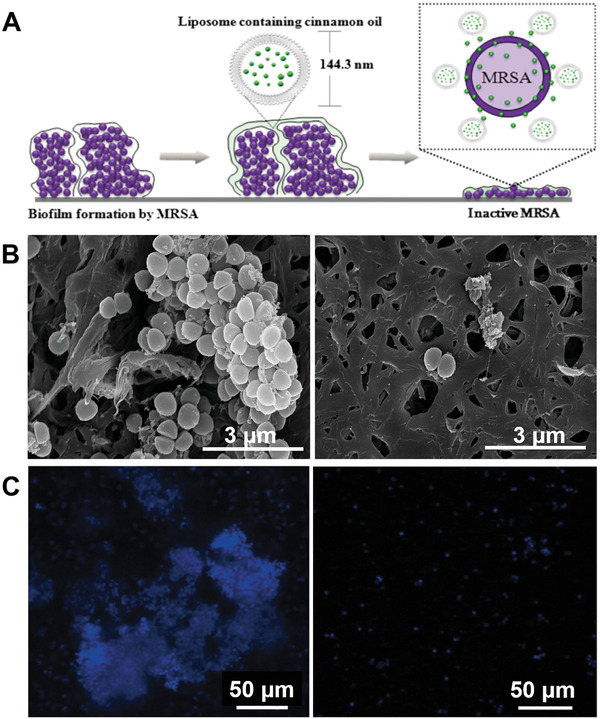
Liposomes containing cinnamon oil against MRSA biofilms. A) Schematic illustrating liposomes containing cinnamon oil with anti‐biofilm activity against MRSA biofilms. B) SEM of MRSA biofilms before and after the treatment of liposomes containing cinnamon oil. C) CLSM images of MRSA biofilms before and after the treatment of liposomes containing cinnamon oil. Reproduced with permission.^[^ ^331^ ^]^ Copyright 2016, Taylor & Francis Group.

Liposomes are composed of phospholipid bilayers which are compatible vesicles and stand for one of the most studied organic NPs for antimicrobial agent delivery.^[^ ^327^ ^]^ Antimicrobial agents loaded liposomes are able to penetrate biofilms, exhibiting high efficacy in treating biofilms composed of many types of bacterial species.^[^ ^328^ ^]^ Many researchers focus on using liposomes to deliver or enhance antibiofilm agents. For example, Giordani et al. incorporated a biosurfactant extracted from *Lactobacillus gasseri* BC9 into liposomes and then demonstrated their capability in inhibiting the biofilm establishment and removing MRSA biofilms.^[^ ^329^ ^]^ In particular, biosurfactant‐incorporated liposomes exhibited better efficacy than free biosurfactants in preventing MRSA biofilm formation. To improve the bioavailability, dissolution, and cellular uptake, Bhatia et al. encapsulated berberine and curcumin in liposomes and demonstrated co‐encapsulated liposomes can more effectively hamper the growth of MRSA and inhibited the establishment of biofilms when compared to free drugs.^[^ ^330^ ^]^ Additionally, Cui et al. loaded cinnamon oil, a natural and safe spice, in liposomes to increase the stability and examined their antibiofilm efficacy against MRSA on different substrates (e.g., gauze, stainless steel, fabrics, and nylon membrane) by quantification of CFU count (**Figure** **9**A).^[^ ^331^ ^]^ The changes of MRSA biofilms in morphology were examined by SEM and CLSM after treatment with liposomes containing cinnamon oil. Figure 9B shows an SEM image, indicating the biofilms attached to the nylon membrane without treatment appeared to be congested spheres. After treatment with liposomes containing cinnamon oil, an evident reduction in terms of the thickness and size of biofilms was observed. The CLSM images showed much lower fluorescent intensities for the biofilms after administration with cinnamon oil‐loaded liposomes when compared to the control samples (Figure 9C). It seems that 1.0 mg mL^−1^ of cinnamon oil‐loaded liposomes could destruct the MRSA biofilm. All these studies demonstrated that liposome formulations could enhance the stability of various antimicrobial drugs and prolong their action time. To better compare these four most common types of nanomaterials used for combating biofilms, we list their advantages and drawbacks in **Table** **2**.

**Table 2:** The advantages and shortcomings of the four most common types of nanomaterials for combating biofilms

Nanomaterials	Advantages	Shortcomings	Refs.
Metal (Oxide) Nanoparticles	* Controllable long‐term stability *Long‐lasting drug release *High surface‐to‐volume ratio *Abundantly available and has the ability to adapt to extreme conditions.	*Potential toxicity of long‐lasting exposure *Low specificity to the target tissues	[280, 281, 282]
Carbon‐based Nanomaterials	*Excellent physicochemical properties and structural characteristics *Environmentally benign nature *Good biocompatibility	*High production costs *Limited in penetrate and target eradicating biofilms.	[283, 284]
Polymer‐based Nanoparticles	*As drug carriers that deliver the antibiofilm molecules *Flexible structures and predictable kinetics have aided the nanoparticle penetration *Stability against high temperature, enzymatic or microbial degradations	*Biosafety, cytotoxicity, and hemolytic activity, especially those with positively charged surfaces	[285, 286]
Lipid‐based Nanoparticles	*Could incorporate with other antibiofilm drugs *Could encapsulate hydrophilic and lipophilic compounds in the same structure *Targeting ability *Cytotoxicity reduction of the antimicrobials as compared with their free form	*Low retention time can imply higher doses over time	[287, 288]

##### Antimicrobial Peptides

The use of AMPs as a substitute for antibiotics has sparked great interest in the past twenty years, and more recently AMPs have been applied for combating microbial biofilms.^[^ ^332^ ^]^ AMPs (over 3000 in the updated AMP database https://www.aps.unmc.edu) are broad and can fold into a variety of structures, ranging from linear to cyclic scaffolds.^[^ ^333^ ^]^ Many linear peptides become amphipathic helix, which is ideal to permeabilize bacterial membranes. Such a property makes them uniquely effective in the rapid killing of multidrug‐resistant bacteria. Hence, they are effective against both dormant and growing cells, irrespective of the cells’ metabolic state.^[^ ^334^ ^]^ The positive charge of AMPs promotes preferential interaction with negatively charged bacterial surfaces rather than host cells.^[^ ^335^ ^]^ However, several disadvantages of such linear peptides limited their clinical applications, such as high instability because of the fast degradation and lysis by proteases.^[^ ^336^ ^]^ However, some cyclic peptides (e.g., vancomycin and daptomycin) have already been used clinically.^[^ ^337^ ^]^ Currently, many scientists are committed to developing synthetic/engineered AMPs to overcome the disadvantages of linear AMPs and improving the production of new cyclic peptides to make them more efficient or available in the field of biofilm elimination.

Cathelicidin‐derived peptides exert potent antimicrobial and antibiofilm activity against many species of multi‐drug resistance pathogens. For example, Pompilio et al. examined the antibiofilm efficacy of multiple peptides stemming from cathelicidin in the treatment of *S. aureus*, *P. aeruginosa*, and *S. maltophilia* strains that were obtained from patients with CF.^[^ ^338^ ^]^ It was found that established biofilms were dramatically influenced by sheep myeloid AMPs‐27/28/29 in their bactericidal concentrations. This study suggests the possibility of using cathelicidin‐derived peptides as therapeutics for the management of CF lung disease. Blower et al. reported the cathelicidin peptides (e.g., LL‐37, NA‐CATH, and SMAP‐29) were effective to inhibit the *Burkholderia thailandensis* biofilm formation.^[^ ^339^ ^]^ They also demonstrated that preformed biofilms could be dispersed by LL‐37 and its D‐enantiomer D‐LL‐37. Mishra et al. discovered that the major AMP (GF‐17) of LL‐37 and its engineered peptide (17BIPHE2) were more effective than their parent peptide in disrupting preformed biofilms of MRSA.^[^ ^340^ ^]^ In the case of clinical *P. aeruginosa*, a combination of 17BIPHE2 with conventional antibiotics was more effective in disrupting preformed biofilms.^[^ ^128^ ^]^ In addition, LL‐37 engineered peptides with stability to proteases prevented biofilm growth on a catheter implanted in murine.^[^ ^341^, ^342^ ^]^ It appears that longer LL‐37 peptides have a certain advantage in biofilm disruption.^[^ ^343^ ^]^

**Figure 10:**
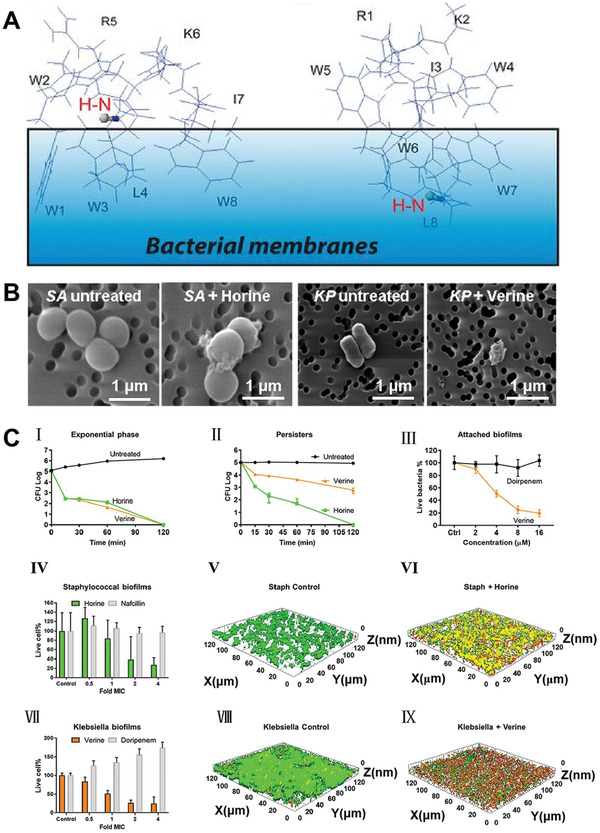
Two distinct amphipathic peptide antibiotics with antibiofilm efficacy. A) N‐labeled leucine in horine (K) and verine‐L (L) indicates an H‐N vector (red) parallel to membrane surface, which allows to position the 3D structure of horine (left) and verine‐L (right) on the lipid bilayer so that the H‐N vector (in ball‐and‐stick) is approximately parallel to the bacterial membranes. B) SEM of *S. aureus* and *K. pneumoniae* before and after the AMP treatment. C) in vitro antibiofilm efficacy of horine (green) and verine (gold). I): Horine and verine (2 × MIC) killed the exponential phase *S. aureus* USA300 LAC in 120 min. II): The two peptides (2 × MIC) also killed nafcillin‐induced persisters of *S. aureus*. III): Verine, but not doripenem, inhibited the attachment of *K. pneumoniae* E406‐17 in a dose‐dependent manner. IV): Horine disrupted the *S. aureus* biofilms established in 48 h. In the confocal images, live bacteria in the untreated control are in V) green and VI) dead bacteria treated by 16 µm of horine are in red. VII): Verine was effective in disrupting the *Klebsiella* biofilms established in 48 h. In the confocal images, live *K. pneumoniae* are in green, and dead *K. pneumoniae* are in IX) red. Reproduced with permission.^[^ ^347^ ^]^ Copyright 2020, National Academy of Science.

Studies have reported AMPs and the modification of their sequence to achieve novel antibiofilm therapeutics with enhanced efficacy and more stability against proteolytic lysis. For example, Thankappan et al. fabricated a helical cationic peptide KABT‐AMP with the sequence GIWKKWIKKWLKKLLKKLWKKG, showing wide‐spectrum antimicrobial activity to kill different bacterial and fungal strains.^[^ ^344^ ^]^ The peptide was also effective to treat the *Candida tropicalis* (a clinical isolate) biofilm. Cardoso et al. reported an *Escherichia coli*‐derived AMP (EcDBS1R5) created by pattern recognition and subsequent sequence optimization.^[^ ^345^ ^]^ Two to 32 µm EcDBS1R5 was able to hamper the growth of different strains including Gram‐positive and Gram‐negative, susceptible, and resistant bacteria. Moreover, EcDBS1R5 at a concentration of 16 µm was effective to treat established *P. aeruginosa* biofilms by reducing the survival rate of biofilm‐forming bacteria. The antibacterial and antibiofilm efficacy of EcDBS1R5 was further demonstrated by a 2‐log reduction of *P. aeruginosa* CFU count in a scarification skin infection mouse model. Zapotoczna et al. synthesized an AMP which could be used as a catheter lock solution for combating *S. aureus* biofilm infections.^[^ ^346^ ^]^ The results revealed that the D‐Bac8c^2, 5Leu^ variant was very efficient in eliminating *S. aureus* biofilms on the central venous catheter in rats. It is worth mentioning that Narayana et al. designed two potent amphipathic short peptides based on database guidance and structure theory (**Figure** **10**).^[^ ^347^ ^]^ These two peptides had the same amino acid sequence exhibiting two different amphipathic structures including a typical horizontal helix (horine) and a new vertical spiral structure (verine) upon binding to membranes. The solid‐state 15N NMR data indicated the horizontal and vertical orientations of peptides on membranes (Figure 10A). Multi‐drug resistant bacteria covering most ESKAPE pathogens were used to examine the potency of horine and verine. These two peptides were effective in treating gram‐positive pathogens including VRE and MRSA. The bacterial damage was characterized by SEM. The *S. aureus* surface was smooth without treatment. Contrarily, the membrane blebs were observed after horine treatment. The *K. pneumoniae* surface was completely destructed after incubation with verine (Figure 10B). *S. aureus* USA300 LAC and persisters were rapidly destroyed when treated with peptides at double concentrations of MIC (Figure 10C (I), (II)). Their efficacy against biofilms was further assessed that are extremely hard to remove using traditional antibiotics. Verine was found to hamper the adherence of *K. pneumoniae* (Figure 10C (III)). Besides, these peptides also showed activity against the established biofilms. Horine showed efficacy in the treatment of *S. aureus* USA300 biofilms in a concentration‐dependent manner (Figure 10C (IV), green), but nafcillin was ineffective (Figure 10C (IV), gray). Similarly, verine destructed the established *K. pneumoniae* biofilms (Figure 10C (VII), gold). In contrast, doripenem was not effective against biofilms in the same experiment (Figure 10C (VII), gray). These two peptides can kill the pathogens in biofilms as shown by CLSM images. Bacteria were living in green without treatment (Figure 10C (V), (VIII)), while bacteria were dead in red after treatment (Figure 10C (VI), (IX)). This work represents a step forward to acquiring new antibiotics as majority of AMPs developed so far are mainly promising for topical treatment of infections and the peptides developed in this work greatly enhanced the survival rate of mice with sepsis and eliminated the infections associated with major organs in the body after one shot without exerting any evident negative effects.

##### Combined Chemical and Physical Approach: Antimicrobial Microneedles

The microneedle patch is a device consisting of a membrane and an array of microscale needles on the surface which was initially invented for transdermal drug delivery. Contrasting to injections using traditional hypodermic needles, microneedle patches are normally considered as a low‐cost, localized, and minimally invasive drug administration approach.^[^ ^348^ ^]^ Microneedles can assist drugs to penetrate the major diffusion skin barrier stratum corneum or directly deliver drugs to the microcapillary in the dermis by simply varying the geometric dimensions of microneedles or tailoring the force applied to the patch. This technology has been broadly investigated since its first appearance in 1998 for many applications including vaccination, anti‐diabetic therapies, anti‐obesity therapies, anti‐inflammatory therapies, local anesthesia, contraception, and anti‐tumor therapies.^[^ ^349^ ^]^ Based on a similar principle, microneedles could readily penetrate the major diffusion barrier EPS of biofilms for delivery of various antimicrobial agents.

To overcome the shortcomings (e.g., poor penetration, off‐target side effects, and induction of antibiotic resistance) of current treatment approaches, Xu et al. showed a microneedle patch for effective treatment of biofilms through enhancing the penetration into the biofilm and precisely delivering antibiotics to specific locations.^[^ ^350^ ^]^ The patches consisted of dissolvable microneedle arrays where the needle tips were incorporated with chloramphenicol‐loaded, gelatinase‐responsive gelatin nanoparticles. The hypotheses were that the microneedles can physically pass through the EPS and after the rapid dissolution of the microneedles, the gelatin NPs can release encapsulated chloramphenicol within the biofilm matrix upon exposure to gelatinase produced by resident microbe. The microneedle system appeared to be more effective than free drugs in terms of the treatment of *Vibrio vulnificus* biofilms. However, this work was limited to the use of microneedle patches for treating biofilms in vitro. In another study, Woodhouse et al. generated a flexible microneedle array patch that was able to simultaneously deliver oxygen and antimicrobial agents.^[^ ^351^ ^]^ The microneedle patches consisted of needle arrays made of polyvinylpyrrolidone and calcium peroxide and a flexible polyethylene terephthalate substrate. Such patches can raise the oxygen contents from 8 to 12 ppm in the first 2 h after administration meanwhile showing high efficacy in treating one‐week‐old biofilms normally seen in skin wounds. This study further demonstrated the penetration of the microneedle arrays and effective management of biofilms in an ex vivo porcine wound infection model. Similarly, Permana et al. demonstrated a dissolvable microneedle array patch with the incorporation of bacterial responsive doxycycline‐loaded NPs for promoting biofilm penetration and delivering doxycycline to the infected areas.^[^ ^352^ ^]^ Such a patch was able to reduce the bacterial bioburdens by about 99.99% in an ex vivo porcine skin biofilm model after being applied for 48 h.

**Figure 11:**
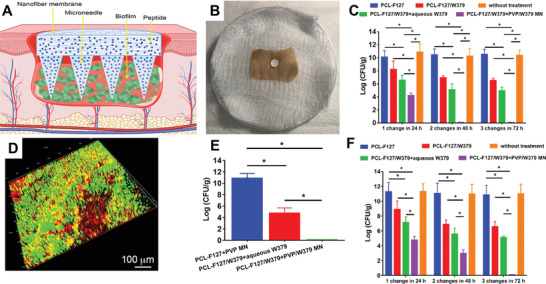
Microneedle‐based dressings for eradication of biofilms on skin wounds. A) Schematic illustrating Janus‐type antimicrobial dressings consisting of molecularly engineered peptide‐loaded electrospun nanofiber membranes and microneedle arrays for the treatment of biofilms in chronic wounds. B) Photograph showing the biofilm treatment by Janus‐type antimicrobial dressings in an ex vivo biofilm‐containing human skin wound model. C) The antibiofilm efficacy test of Janus‐type dressings in an ex vivo human skin wound model. The dressings were changed three times every 24 h. D) Live/dead staining for the tissue collected from wounds after 24 h of MRSA inoculation and subsequent 24 h of 2% mupirocin treatment, indicating the biofilm formation in type II diabetic mice wounds. E) Antibiofilm efficacy of Janus‐type antimicrobial dressings in vivo. The dressings were changed three times every 24 h. F) Antibiofilm efficacy of Janus‐type dressings against *P. aeruginosa*/MRSA blend biofilms in an ex vivo human skin wound model. The dressings were changed three times every 24 h. PCL‐F127: PCL‐F127 nanofibers. PCLF127/W379: W379 peptide‐loaded PCL‐F127 nanofibers. PCL‐F127/W379+aqueous W379: W379 peptide‐loaded PCL‐F127 nanofibers + free W379 peptides. PCL‐F127/W379+PVP/W379 MN: Janus‐type dressing composed of W379 peptide‐loaded PCL‐F127 nanofiber membrane and W379 peptide‐loaded microneedle arrays. Without treatment: no treatment for the wounds. Reproduced with permission.^[^ ^353^ ^]^ Copyright 2020, American Chemical Society.

It is known that sometimes biofilms regrow within days after treatment. In light of this, Su et al. reported a novel Janus‐type dressing consisting of molecularly engineered AMPs‐loaded dissolvable microneedles arrays and electrospun nanofiber membranes serving as a substrate.^[^ ^353^ ^]^ It was believed that microneedle arrays can physically pass through biofilms and release AMPs within biofilms and electrospun nanofiber membranes can release peptides to the outside of biofilms. The released peptides from both needles and nanofibers could cause biofilm removal initially. In comparison, the sustained‐release peptides from nanofiber membranes may be able to prevent the recurrence of biofilms. The dressing, comprising of nanofiber substrate and water‐soluble microneedle arrays, allowed precise delivery of AMPs to both interior and exterior of the biofilms (**Figure** **11**A). Notably, this dressing can eliminate MRSA biofilms formed in ex vivo human skin wounds (Figure 11B,C) and type II diabetic mice wounds (Figure 11D,E) after administration day by day without performing debridement. In addition, the dressing could get rid of the biofilms consisting of *P. aeruginosa* and MRSA binary strains established in ex vivo human skin wounds as well (Figure 11F). However, the engineered AMPs are considered new chemical entities during the regulatory approval process. Because of the attrition rate of the current drug discovery model, high cost, and time‐consuming, it is reasonable to reformulate the current antimicrobial agents used in clinics for the effective management of biofilms. Thus, Su et al. attempted to incorporate multiple antimicrobial agents (e.g., AgNO_3_, Ga(NO_3_)_3_, and vancomycin) that target bacteria with different modes of action into a biphasic dressing similar to the above‐described comprising of water‐soluble microneedle arrays and electrospun nanofiber membranes for effective treatment of biofilms.^[^ ^354^ ^]^ The dressings can remove both MRSA and MRSA/*P. aeruginosa* blend biofilms formed in ex vivo human skin wounds without using debridement. These studies were primarily focused on the treatment of biofilms in wounds. Microneedle arrays could attach to 3D scaffolds to generate functional wound dressings capable of effectively delivering multiple distinct molecules such as antimicrobial agents, immunomodulating compounds, and growth factors for the elimination of biofilms in wounds and promotion of healing. Taken together, these recent developments add flexibility to antibiofilm treatments depending on the nature of the resistant pathogens. A combination of AMPs with existing antimicrobial agents, especially via the co‐delivery device, holds a great promise for eradicating difficult‐to‐treat biofilms containing pathogens that cannot be killed by traditional antibiotics alone.

## 5 Future Directions

Despite great strides have been made because of the discovery of many antibiofilm agents and strategies, the biofilm‐related infections remain to be a public health problem. In order to develop more effective strategies for combating biofilms, it is necessary to better understand the biology of biofilms which will eventually benefit the identification of new targets for the eradication of biofilms. Current in vitro biofilm models are mainly established by culturing bacteria on different substrates. Because of the convenience, we anticipate the continued use of microplates in biofilm research. In rich media, the formation of biofilms is time‐dependent and indicated by a constant level of biomass.^[^ ^128^ ^]^ It allows the analysis of biofilms by different staining methods (e.g., crystal violet and XTT) for additional insight.^[^ ^129^ ^]^ The results obtained in such a static model can be similar to those obtained in a fluidic biofilm model.^[^ ^340^ ^]^ Additional knowledge can be obtained by developing new biofilm models. For example, a better in vitro biofilm model could be developed by printing bacteria and hydrogels which will be capable of mimicking native biofilms.^[^ ^355^, ^356^, ^357^, ^358^, ^359^ ^]^

In order to develop more effective approaches for managing biofilms, future studies should aim to identify new targets for disrupting biofilms. Furthermore, the advances in genetic tools and resources (e.g., genome‐wide transposon insertion libraries) may provide a high‐throughput screening platform to find out new targets among the non‐essential genes.^[^ ^360^ ^]^ In combination with new technologies (e.g., CRISPR interference (CRISPRi) knockdown), it may be possible to better understand the molecular mechanisms of biofilm‐related functions (e.g., identifying genes linked with the release of eDNA).^[^ ^361^ ^]^ The discovered new targets will be important to assist in developing new antibiofilm agents and strategies, including a combined use with inhibitors with existing antibiotics.

In addition, other than interactions between bacteria within biofilms, the bacteria within biofilms use electrical signaling to recruit distant motile cells in a species‐independent manner to expand biofilms.^[^ ^362^ ^]^ Molecules or methods to disrupt the electrical signaling within biofilms could be used to inhibit their expansion and formation. Combinatorial therapies are promising for biofilm treatment. Use of combinations of antimicrobial agents with distinct acting mechanisms normally provides the best synergistic effect in both bacterial killing and the reduction of drug resistance development.^[^ ^363^ ^]^ The antibiotic‐resistant bacteria could be sensitive to combinatory therapies, while mono‐therapies may not be effective.^[^ ^364^ ^]^ This approach could be vital to reuse many antibiotics otherwise useless because of multidrug resistance although the molecular mechanism is not fully understood.

Although many delivery systems have been developed for combating biofilms, more efforts should be devoted to the development of on‐demand antimicrobial drug delivery systems by integrating soft electronic devices with biosensors.^[^ ^365^, ^366^ ^]^ In this way, it may be possible to avoid the unnecessary delivery or overdose of antibiotics and eventually reduce the chance to develop drug resistance. In addition, more studies should be carried out to discover more reliable biomarkers which can be used to sense infection at an early stage. Additionally, triggering biofilm‐disruptive activity in response to pathogenic micro‐environments (e.g., acidic pH, hypoxia, temperature, and pathogen‐derived metabolites) and targeting bacteria specifically (e.g., bacterial exotoxins, outer membrane proteins, d‐amino acids of cell membrane, and competitive displacement) could be other interesting directions.^[^ ^367^, ^368^, ^369^ ^]^ The ultimate goal may concentrate on the development of doable and affordable dosage forms and collaborations with industrial partners to achieve the highest potency and safety with long‐lasting therapeutic effects and minimal cytotoxicity for use in clinics.

## Conflict of Interest

The authors declare no conflict of interest.
